# Supplementary material for: Compartmentalized Epidermal Activation of β-Catenin Differentially Affects Lineage Reprogramming and Underlies Tumor Heterogeneity
Source: Cell Rep. 2016 Jan 7;14(2):269–81. doi: 10.1016/j.celrep.2015.12.041 (PMC4713864; doi:10.1016/j.celrep.2015.12.041)
Supplement: Document S1. Supplemental Experimental Procedures and Figures S1–S7 [file mmc1.pdf]

SUPPLEMENTAL DATA ITEMS

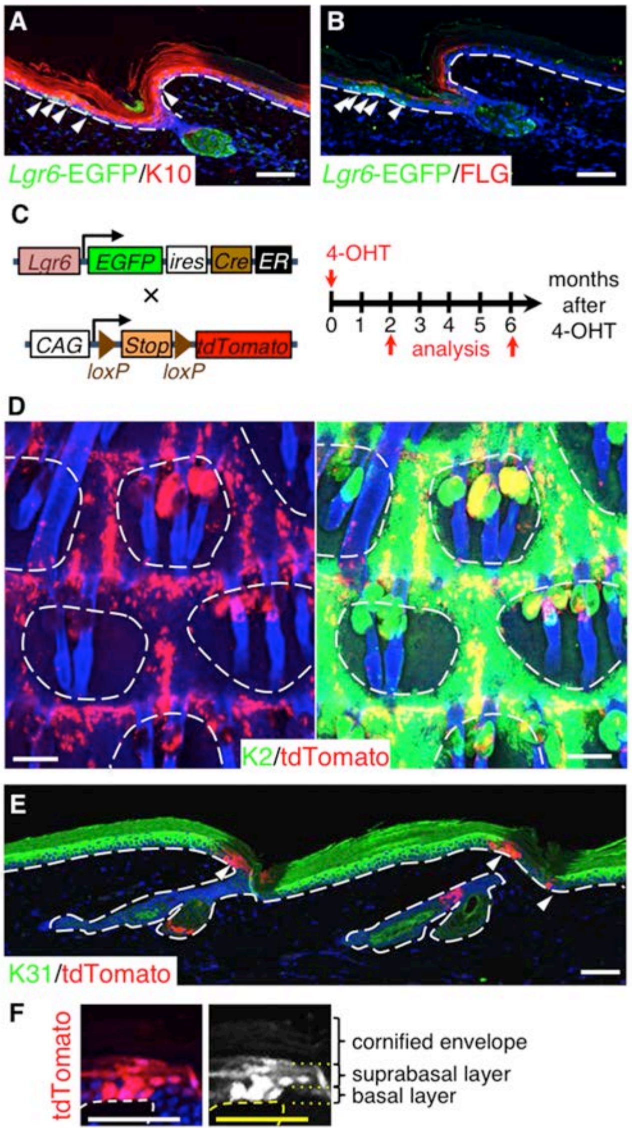

Figure S1 (related to Figure 1). *Lgr6*-expressing cells in the interfollicular epidermis give rise to the interscale differentiated lineage. (A, B) Tail skin sections collected from adult *Lgr6* KI mice stained with anti-EGFP (green), anti-K10 (red in A), anti-filaggrin (FLG; red in B) and counterstained with DAPI to label nuclei (blue). **(C)** Schematic representation of the genetic elements for lineage tracing during adult homeostasis and the experimental set-up. **(D)** Tail epidermal wholemounts collected from *Lgr6* KI/*R26R-tdTomato* mice collected 6 months after 4-OHT treatment, stained with anti-tdTomato (in red) and anti-K2 (in green). Wholemounts were counterstained with DAPI to label nuclei or with phalloidin to label F-actin (blue). Dashed lines demarcate IFE scales. **(E, F)** Tail skin sections collected from *Lgr6* KI/*R26R-tdTomato* mice twelve weeks after 4-OHT treatment, stained with anti-tdTomato (red) and anti-K31 (green) and counterstained with DAPI to label nuclei (blue). Dashed lines indicate epidermal-dermal boundary. Arrowheads denote $LGR6^{+}$ cells or their progeny. F: higher magnification views of tdTomato+ IFE cells in (E). Scale bars: 100 μm (A, B, E, F), 50 μm (D).

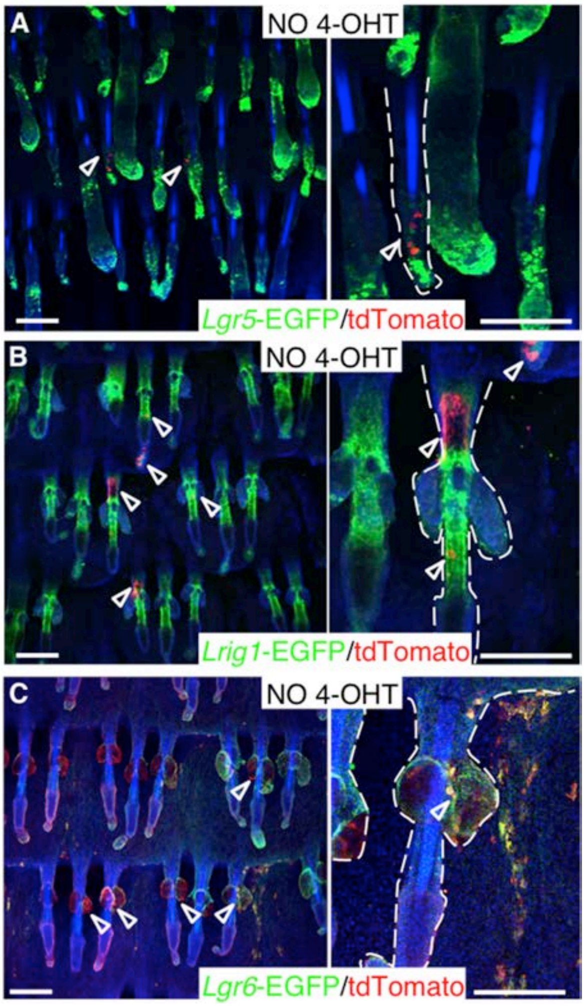

Figure S2 (related to Figure 1). Leakiness of KI reporters. (A) Detection of *Lgr5*-EGFP (green) and tdTomato (red) in tail epidermal wholemounts of acetone-only treated adult *Lgr5* KI/*R26R-tdTomato* mice. **(B)** Detection of *Lrig1*-EGFP (green) and tdTomato (red) in tail epidermal wholemounts of acetone-only treated adult *Lrig1* KI/*R26R-tdTomato* mice. **(C)** Detection of *Lgr6*-EGFP (green) and tdTomato (red) in tail epidermal wholemounts of acetone-only treated adult *Lgr6* KI/*R26R-tdTomato* mice. Dashed lines indicate pilosebaceous units. Arrowheads: $tdTomato^+$ cells. Scale bars: 100 µm.

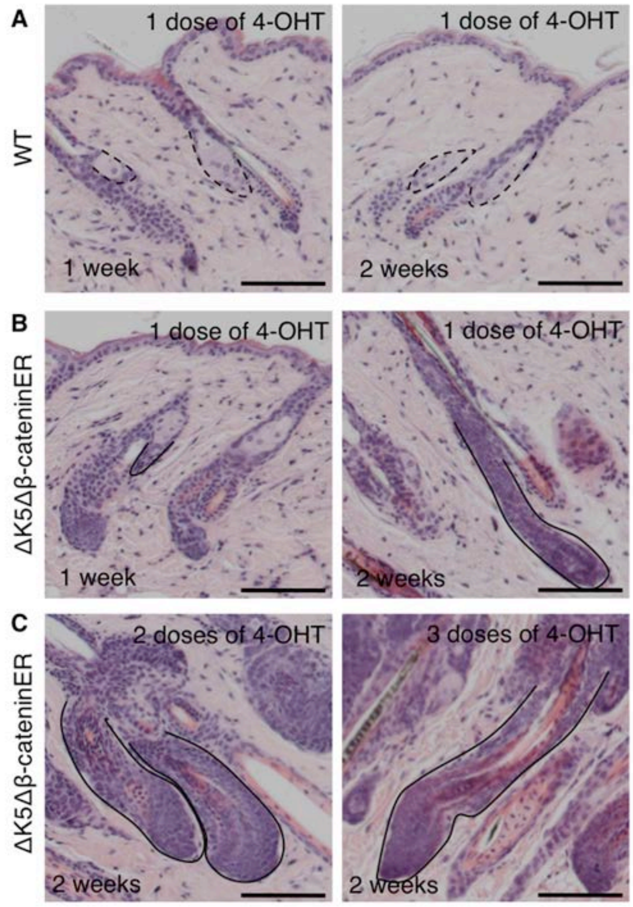

Figure S3 (related to Figure 2). Optimization of 4-OHT dose and tissue collection time points for ectopic hair follicle formation. (**A-C**) Wild-type (A) or ΔK5ΔNβ-cateninER transgenic mice (B, C) received one to three doses of 1.5 mg 4-OHT and tissue was collected one or two weeks later as indicated. Back skin sections were stained with H&E. Lines indicate ectopic HFs and dashed lines demarcate sebaceous glands. Scale bars: 200 µm.

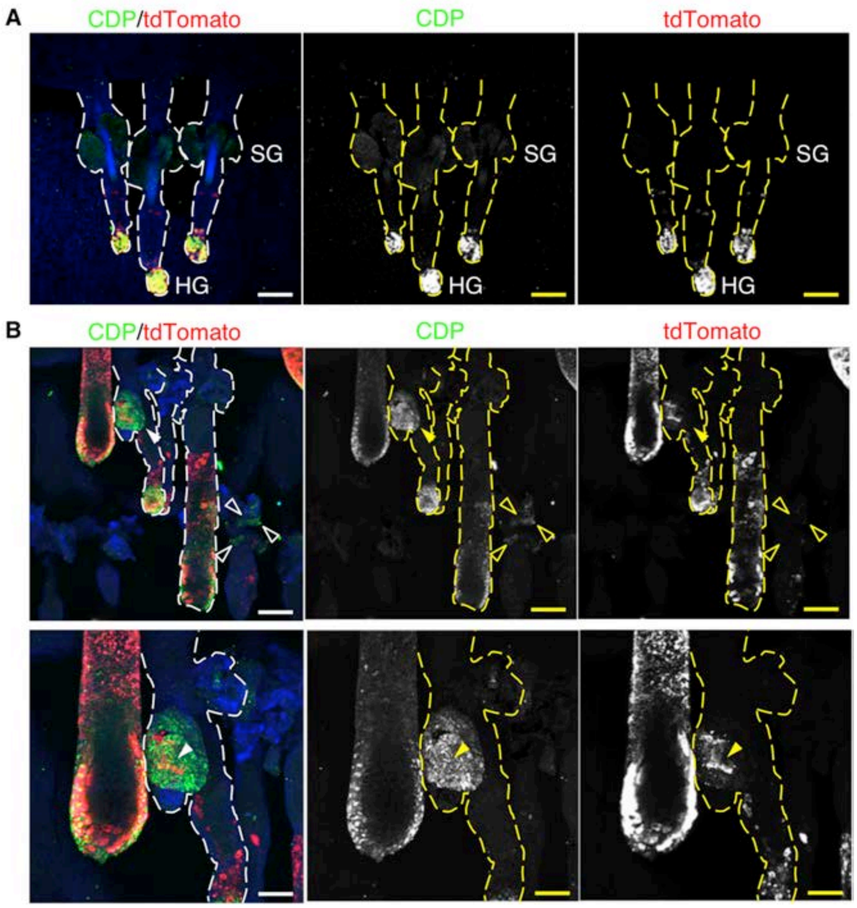

Figure S4 (related to Figure 2). Absence of CDP expression in control tail skin and example of tdTomato[+] progeny of *Lgr5*-expressing hair follicle stem cells. (A, B) Tail epidermal wholemounts collected one week after 4-OHT treatment and stained with anti-tdTomato (red) or anti-CDP (green), as indicated. Wholemounts were counterstained with DAPI to label nuclei or with phalloidin to label F-actin (blue). (A) *Lgr5* KI/*R26R-tdTomato* control mouse; (B) *Lgr5* KI/*R26R-tdTomato*/ΔK5ΔNβ-cateninER transgenic mouse. All panels in A show the same region of epidermis. All panels in B show the same region of epidermis, but lower panels are higher magnification views of upper panels. Single-colour images for individual markers are shown in grey scale. Dashed lines demarcate HF, SG and ectopic HFs. Individual ectopic HFs are indicated with closed arrowheads. Ectopic HF in IFE is shown with open arrowheads. Scale bars: 100 µm (A, upper panels in B), 50 µm (lower panels in B).

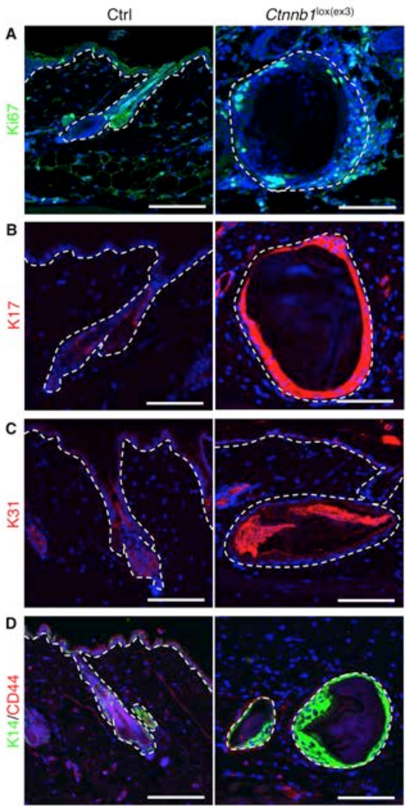

Figure S5 (related to Figure 3). Expression of Ki67, K17, K31 and CD44 following oncogenic β-catenin stabilisation in LGR5^{+} stem cells Paraffin sections of back skin collected from *Lgr5* KI/*R26R-tdTomato*/*Ctnnb1*$^{lox(ex3)/+}$ mutant and control *Lgr5* KI/*R26R-tdTomato*/*Ctnnb1*$^{+/+}$ (Ctrl) mice eight weeks after 4-OHT treatment, stained with antibodies against Ki67 (A), keratin 17 (K17), K31 (C), keratin 14 (D) and CD44 (D), counterstained with DAPI (blue). Dashed lines denote epidermal-dermal boundaries. Scale bars: 200 μm.

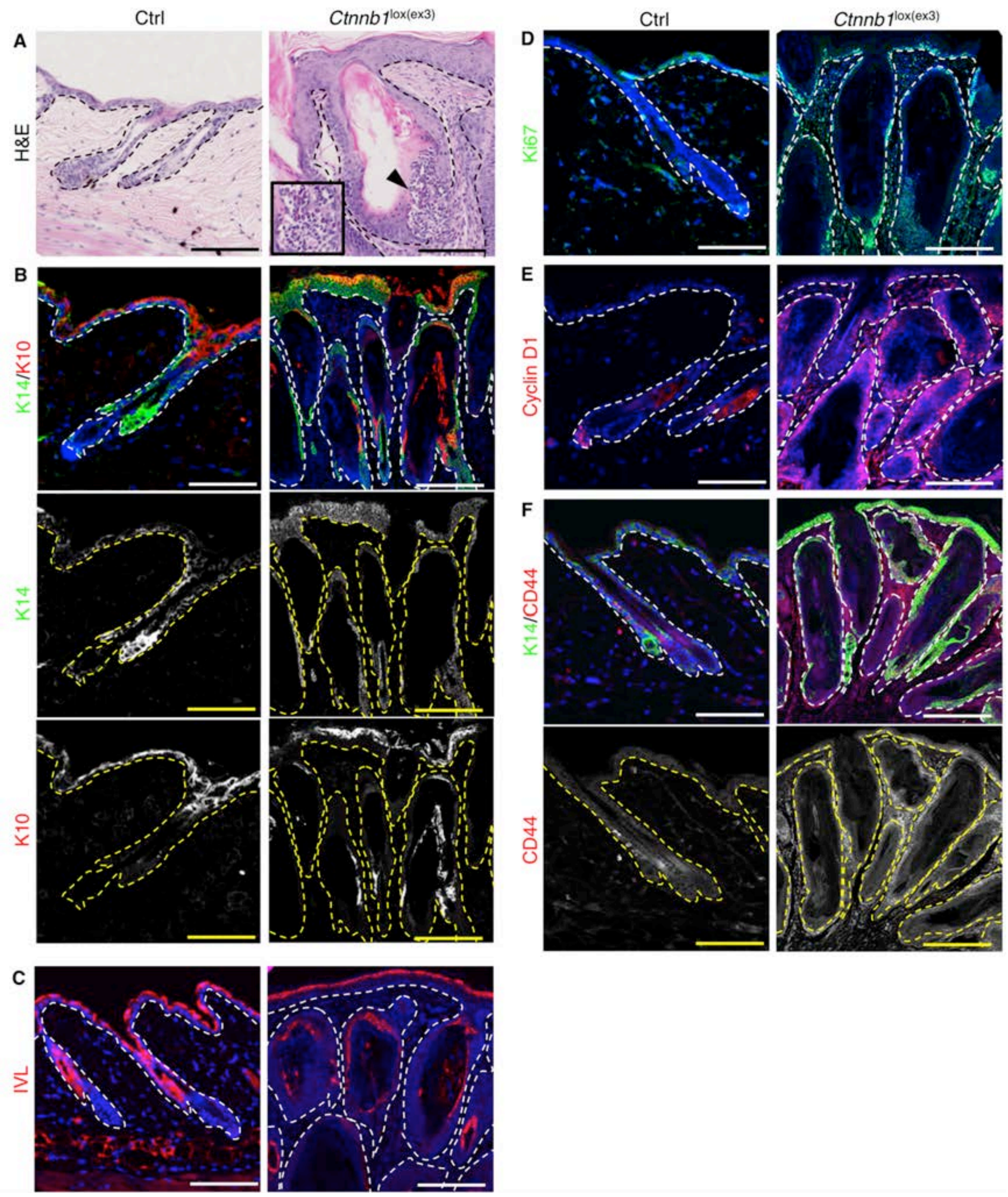

Figure S6 (related to Figure 4). Effects of oncogenic β-catenin stabilisation in LRIG1^{+} stem cells. Paraffin sections of back skin from *Lrig1* KI/*R26R-tdTomato*/*Ctnnb1*$^{lox(ex3)/+}$ mutant and control *Lrig1* KI/*R26R-tdTomato*/*Ctnnb1*$^{+/+}$ (Ctrl) mice four weeks after 4-OHT treatment, stained with H&E (A) or antibodies against K14 (green in B and F), K10 (red in B), involucrin (IVL; red in C), Ki67 (green in D), cyclin D1 (red in E), and CD44 (red in F), counterstained with DAPI (blue). Single-colour images for some markers are shown in grey scale, as indicated. Insert in (A) shows arrowed area at higher magnification. Dashed lines denote epidermal-dermal boundaries. Scale bars: 200 μm.

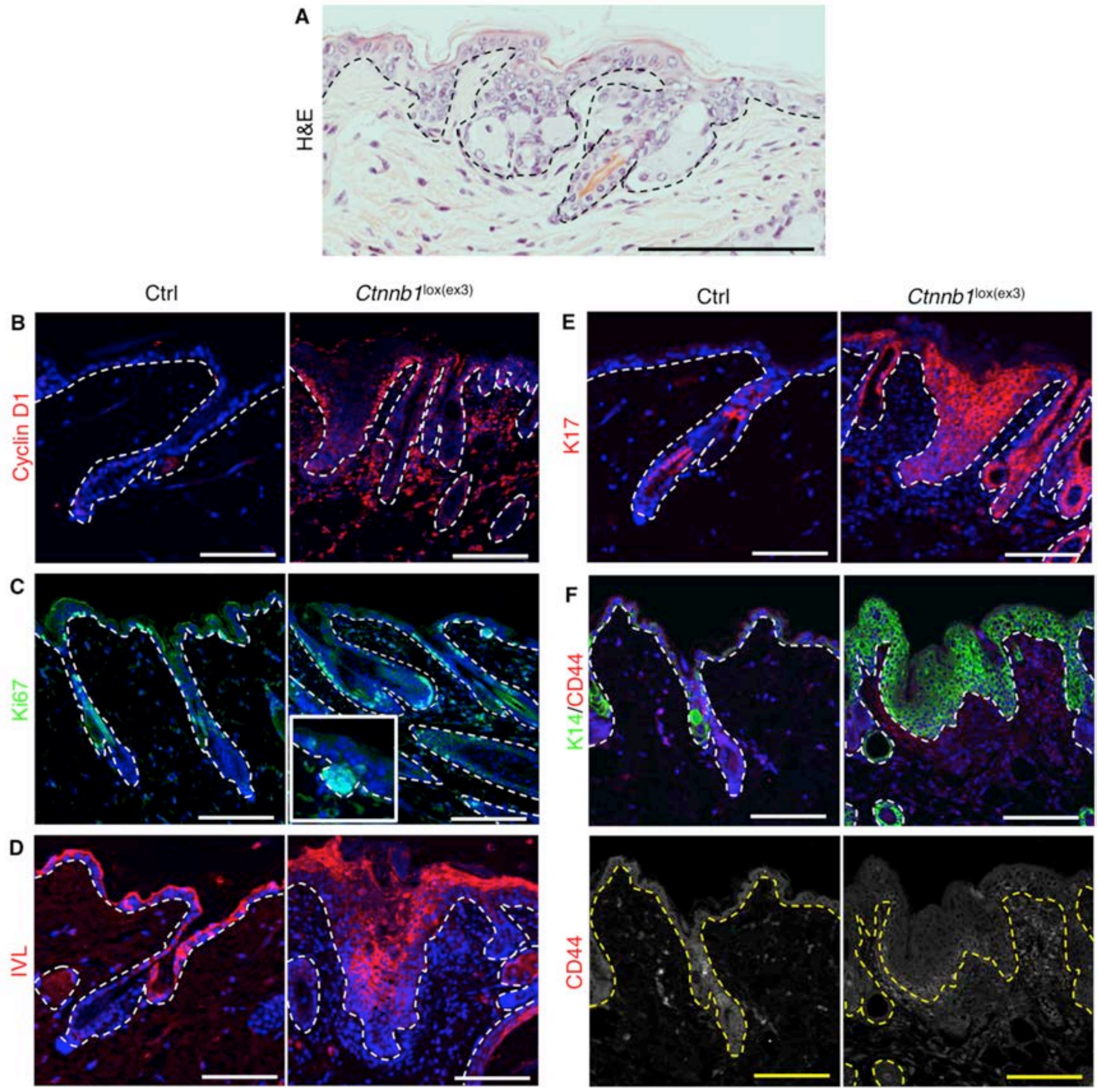

Figure S7 (related to Figure 5). Effects of oncogenic β-catenin stabilisation in LGR6^{+} stem cells. Paraffin sections of back skin collected from *Lgr6* KI/*R26R-tdTomato*/*Ctnnb1*$^{lox(ex3)/+}$ mutant and control *Lgr6* KI/*R26R-tdTomato*/*Ctnnb1*$^{+/+}$ (Ctrl) twelve (A) or eight (B-F) weeks after 4-OHT treatment, stained with H&E (A) or antibodies against cyclin D1 (red in B), Ki67 (green in C), involucrin (IVL; red in D), keratin 17 (K17; red in E), K14 (green in F), CD44 (red in F) and counterstained with DAPI (blue). Single-colour images for CD44 are shown in grey scale (F). Dashed lines denote epidermal-dermal boundaries. Insert in (B) shows IFE down growth (main panel) at higher magnification. Scale bars: 200 µm.

SUPPLEMENTAL EXPERIMENTAL PROCEDURES

Mice

For tdTomato lineage tracing and ectopic HF induction 7-9 week old adult mice were used. 1.5 mg 4-hydroxy-Tamoxifen (4-OHT; Sigma-Aldrich H7904) dissolved in 100 µL acetone (Sigma-Aldrich) was applied to the tail and shaved back skin. Activation of the *Ctnnb1* mutant allele was achieved by topical application of 1.5 mg 4-OHT dissolved in 100 µL acetone to shaved back skin of 7-9 week old adult mice. Both male and female mice were treated in the experiments and similar results were obtained.

Histology and immunohistochemistry

For immunohistochemistry, 5 µm sections were prepared and either stained with haematoxylin and eosin (H&E), stained following Herovici's protocol or subject to conventional antibody staining procedures as described elsewhere (Kretzschmar et al., 2015).

Primary antibodies used were: mouse anti-β-catenin (1:500; BD Bioscience 610153), goat anti-CD26 (1:50; R&D Systems AF1180), rat anti-CD44 (1:100; Abcam ab119863), rat anti-CD45 (1:100; eBioscience 14-0451), goat anti-CDP (1:100; Santa Cruz sc-6327), mouse anti-cyclin D1 (1:100; Santa Cruz sc-450), anti-F4/80 (eBioscience), rabbit anti-Filaggrin (1:100; Abcam ab24584), rabbit anti-GFP (1:500; Life Technologies A11122), chicken anti-GFP (1:500; Abcam ab13970), goat anti-GFP (1:200; Abcam ab6673), rabbit anti-involucrin (1:800; in-house ERLi-3), rabbit anti-K10 (1:100; Covance PRB-159P), mouse anti-K14 (1:1000; in-house LL002), rabbit anti-K14 (1:1000; Covance PRB-155P), chicken anti-K14 (1:1000; Covance SIG-3476), rabbit anti-K17 (1:300; in-house LT3TB4), guinea pig anti-K31 (1:100; Progen GP-hHa1), rabbit anti-Ki67 (1:100; Abcam ab16667), rat anti-Ki67 (1:300; DAKO M7249), rabbit anti-LEF1 (1:100; Cell Signaling #2230), goat anti-LRIG1 (1:100; R&D Systems AF3688), anti-NFκB (eBioscience), rabbit anti-RFP (1:1000; recognising tdTomato; Rockland 600-401-379), and rabbit anti-vimentin (1:100; Cell Signaling #5741). Antibody staining was visualised using appropriate species-specific secondary antibodies conjugated to Alexa Fluor 488, 555, 594 or 647 (1:300; Life Technologies). Slides were mounted using ProLong Gold anti-fade reagent (Life Technologies) containing 4',6-diamidino-2-phenylindole (DAPI; Sigma) as nuclear counterstain.

All fluorescent sections were analysed on a TCS SP5 confocal microscope (Leica) or an A1 confocal microscope (Nikon). All images of H&E and Herovici stained sections were taken on an Axiophot microscope with an AxioCam HRc camera (Zeiss) or on an A1 confocal microscope with a DS-Fi2 camera (Nikon). H&E stained tumour sections were scanned and analysed using Aperio ImageScope software (Leica).

SUPPLEMENTAL REFERENCE

Kretzschmar, K., Cottle, D.L., Schweiger, P.J., and Watt, F.M. (2015). The Androgen Receptor Antagonizes Wnt/beta-Catenin Signaling in Epidermal Stem Cells. The Journal of investigative dermatology.
