# Supplementary material for: Compartmentalized Epidermal Activation of β-Catenin Differentially Affects Lineage Reprogramming and Underlies Tumor Heterogeneity
Source: Cell Rep. 2016 Jan 7;14(2):269–81. doi: 10.1016/j.celrep.2015.12.041 (PMC4713864; doi:10.1016/j.celrep.2015.12.041)
Supplement: Document S2. Article plus Supplemental Information [file mmc2.pdf]

Article

Cell Reports

Compartmentalized Epidermal Activation of β-Catenin Differentially Affects Lineage Reprogramming and Underlies Tumor Heterogeneity

Graphical Abstract

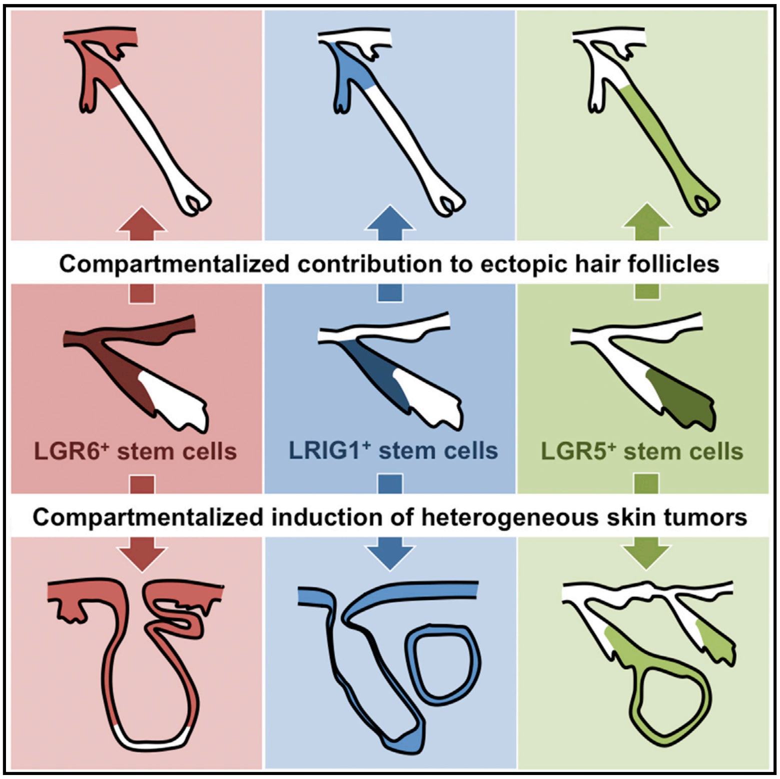

Authors

Kai Kretzschmar, Christine Weber, Ryan R. Driskell, Eduardo Calonje, Fiona M. Watt

Correspondence

fiona.watt@kcl.ac.uk

In Brief

Kretzschmar et al. show that activation of β-catenin in LGR5-, LRIG1-, and LGR6-positive epidermal stem cells elicits different responses in terms of formation of ectopic hair follicles and tumors. Different stem cells are associated with different tumor types and stromal responses. Therefore, compartmentalization of epidermal stem cells underlies tumor heterogeneity.

Highlights

- $LGR6^{+}$ and $LRIG1^{+}$ stem cells give rise to β-catenin-induced hair follicles
- LGR6 defines stem cells of tail interscale interfollicular epidermis
- β-Catenin activation in different stem cells induces distinct tumor types
- Different β-catenin-induced tumors differ in stromal composition

Kretzschmar et al., 2016, Cell Reports *14*, 269–281
January 12, 2016 ©2016 The Authors
http://dx.doi.org/10.1016/j.celrep.2015.12.041

OPEN ACCESS
CellPress

Compartmentalized Epidermal Activation of β-Catenin Differentially Affects Lineage Reprogramming and Underlies Tumor Heterogeneity

Kai Kretzschmar,[1,2,3,5] Christine Weber,[1,3] Ryan R. Driskell,[1] Eduardo Calonje,[4] and Fiona M. Watt[1,*]
[1]Centre for Stem Cells and Regenerative Medicine, King's College London, 28th Floor, Tower Wing, Guy's Hospital, Great Maze Pond, London SE1 9RT, UK
[2]Wellcome Trust–Medical Research Council Cambridge Stem Cell Institute, University of Cambridge, Tennis Court Road, Cambridge CB2 1QR, UK
[3]Department of Genetics, University of Cambridge, Downing Street, Cambridge CB2 3EH, UK
[4]Dermatopathology Laboratory, St. John's Institute of Dermatology, St. Thomas' Hospital, Westminster Bridge Road, London SE1 7EH, UK
[5]Present address: Hubrecht Institute-KNAW and University Medical Center Utrecht, Uppsalalaan 8, 3584 CT Utrecht, The Netherlands
*Correspondence: fiona.watt@kcl.ac.uk
http://dx.doi.org/10.1016/j.celrep.2015.12.041
This is an open access article under the CC BY license (http://creativecommons.org/licenses/by/4.0/).

SUMMARY

Wnt/β-catenin activation in adult epidermis can induce new hair follicle formation and tumor development. We used lineage tracing to uncover the relative contribution of different stem cell populations. LGR6⁺ and LRIG1⁺ stem cells contributed to ectopic hair follicles formed in the sebaceous gland upon β-catenin activation, whereas LGR5⁺ cells did not. *Lgr6*, but not *Lrig1* or *Lgr5*, was expressed in a subpopulation of interfollicular epidermal cells that were competent to form new hair follicles. Oncogenic β-catenin expression in LGR5⁺ cells led to formation of pilomatricomas, while LRIG1⁺ cells formed trichoadenomas and LGR6⁺ cells formed dermatofibromas. Tumor formation was always accompanied by a local increase in dermal fibroblast density and transient extracellular matrix remodeling. However, each tumor had a distinct stromal signature in terms of immune cell infiltrate and expression of CD26 and CD44. We conclude that compartmentalization of epidermal stem cells underlies different responses to β-catenin and skin tumor heterogeneity.

INTRODUCTION

Multiple epidermal stem cell subpopulations in adult mouse skin have been identified over the last decade (Kretzschmar and Watt, 2014; Solanas and Benitah, 2013). Although they tend to be competent to give rise to all the different epidermal lineages following transplantation, wounding, or genetic modification, under steady-state conditions, they maintain the epidermis in a compartmentalized manner (Page et al., 2013; Watt and Jensen, 2009). Stem cells in the lower pilosebaceous unit (PSU) comprising the hair bulge and germ (responsible for hair regeneration) express *Krt19*, *CD34*, *Lgr5*, and *Gli1* (Blanpain et al., 2004; Brownell et al., 2011; Jaks et al., 2008). Stem cells in the upper PSU, consisting of the hair follicle (HF) isthmus, junctional zone, infundibulum, and sebaceous gland (SG), express *Lrig1*, *Lgr6*, and *Plet1* (Jensen et al., 2009; Nijhof et al., 2006; Page et al., 2013; Snippert et al., 2010). Previous studies have identified *Lgr6*-expressing cells in the interfollicular epidermis (IFE) (Liao and Nguyen, 2014; Page et al., 2013) and a recent study has shown that they have stem cell characteristics (Füllgrabe et al., 2015).

The Wnt pathway plays a crucial role in epidermal development, homeostasis, and cancer (Blanpain et al., 2007; Lim and Nusse, 2013; Watt and Collins, 2008). β-Catenin activation is required for HF morphogenesis during embryonic development and for the growth phase of the hair growth cycle in adult skin (Huelsken et al., 2001; Lim et al., 2013; Lowry et al., 2005; Niemann et al., 2002). Recent work suggests that canonical Wnt signaling is also required for IFE stem cell activation during adult homeostasis, as loss of β-catenin in this compartment causes decreased proliferation of basal layer keratinocytes and IFE thinning (Choi et al., 2013; Lim et al., 2013). In adult skin, transient epidermal activation of β-catenin induces ectopic HFs in SG, HF, and IFE (Baker et al., 2010; Lo Celso et al., 2004; Silva-Vargas et al., 2005). However, the different regions of the skin exhibit differential sensitivity to β-catenin, with SG being most sensitive and the HF bulge being highly insensitive (Baker et al., 2010; Deschene et al., 2014; Silva-Vargas et al., 2005).

In addition to its role in normal epidermis, deregulated Wnt signaling is a feature of epidermal tumors (Klaus and Birchmeier, 2008; Watt and Collins, 2008). In human skin, oncogenic activation of β-catenin occurs in HF tumors known as pilomatricomas (Chan et al., 1999), while mutations in the LEF1 transcription factor that prevent binding of β-catenin are associated with SG tumors (Takeda et al., 2006). Consistent with the human studies, in mouse, sustained activation of β-catenin in all stem cell compartments that express keratin 14 (*Krt14*) results in pilomatricomas and trichofolliculomas (Gat et al., 1998; Lo Celso et al., 2004; Van Mater et al., 2003; Watt and Collins, 2008), while

CrossMark

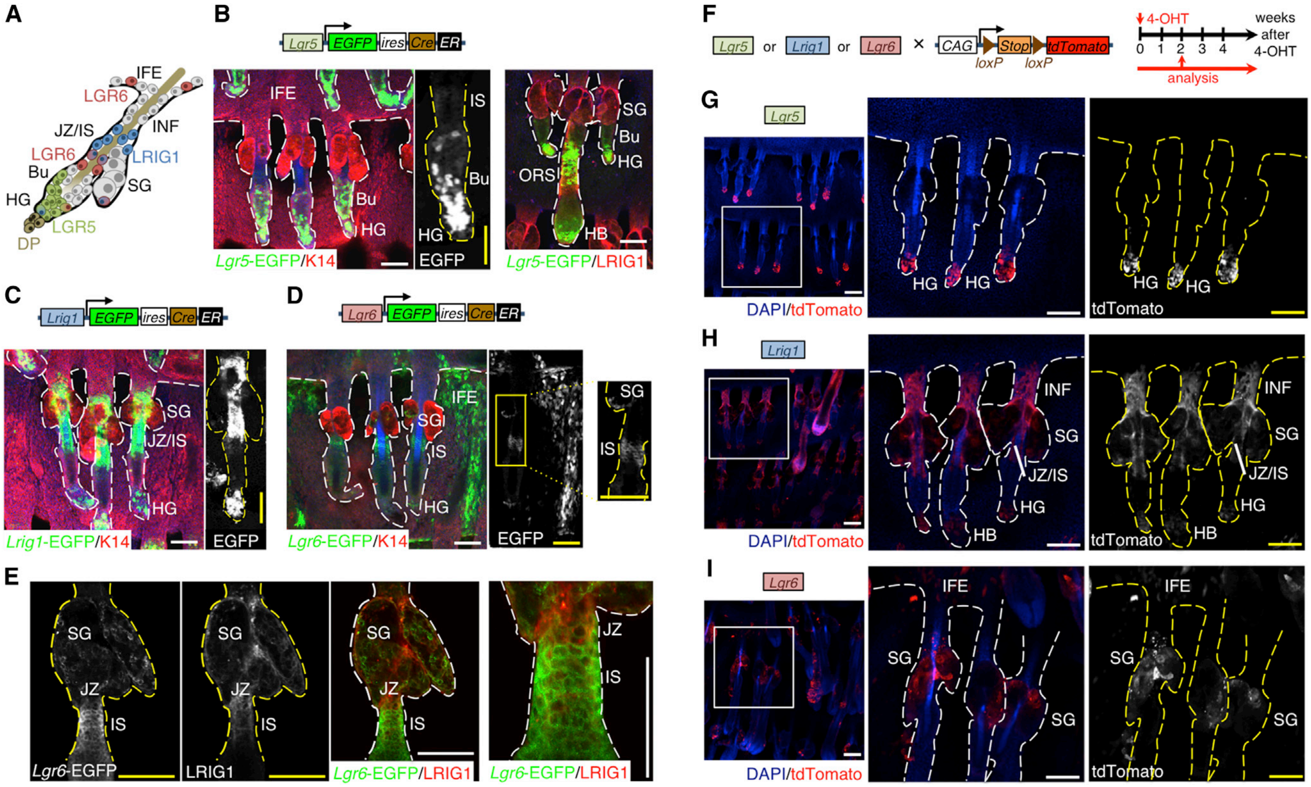

Figure 1. Epidermal Expression and Lineage Tracing of *Lgr5*, *Lrig1*, and *Lgr6* during Adult Homeostasis

(A) Schematic diagram of stem cell marker expression in the epidermis.

(B–E) Detection of *Lgr5*-EGFP (green in B), *Lrig1*-EGFP (green in C), *Lgr6*-EGFP (green in D and E), and K14 (red) or LRIG1 (red) in tail epidermal whole mounts of the respective adult KI mice. Whole mounts were counterstained with phalloidin to label F-actin (blue). Single-color images are shown for some markers in grayscale.

(F) Schematic representation of the genetic elements for lineage tracing during adult homeostasis (control) and the experimental setup.

(G–I) Tail epidermal whole mounts of *Lgr5* KI/*R26R-tdTomato* (G), *Lrig1* KI/*R26R-tdTomato* (H), and *Lgr6* KI/*R26R-tdTomato* (I) mice collected 2 weeks after 4-OHT treatment, stained with anti-tdTomato (red), and counterstained with DAPI to label nuclei (blue). Single-color images for tdTomato are shown in grayscale. Bu, bulge; DP, dermal papilla; HG, hair germ; IFE, interfollicular epidermis; JZ/IS, junctional zone/isthmus; INF, infundibulum; SG, sebaceous gland. Dashed lines demarcate HF, SG, and ectopic HFs. Scale bars, 100 μm.

expression of *ΔNLef1* results in SG tumors (Niemann et al., 2002, 2007). In addition, epidermal deletion of β-catenin causes regression of squamous cell carcinomas (SCCs) (Beronja et al., 2013; Lo Celso et al., 2004; Malanchi et al., 2008). Formation of basal cell carcinomas (BCCs) has also been shown to be dependent on β-catenin activation, either in response to activating mutations in the Hedgehog signaling pathway (Youssef et al., 2010, 2012) or in the absence of vitamin D receptor signaling (Pálmer et al., 2008).

The differential sensitivity of different regions of the epidermis to forming new HFs on transient β-catenin activation and the diversity of epidermal tumor types associated with oncogenic β-catenin mutations raise the question of whether different epidermal stem cell populations exhibit different responses to β-catenin. To investigate this, we activated β-catenin in LGR5$^+$, LGR6$^+$, and LRIG1$^+$ stem cells and examined the consequences. Our results show that the outcome of β-catenin activation is specified by the compartmental origin of the initiating epidermal stem cells and correlates with distinct changes in the underlying dermis.

RESULTS

Expression of *Lgr5*, *Lgr6*, and *Lrig1* in Adult Mouse Epidermis

We used *Lgr5EGFPiresCreER*^{T2} (*Lgr5* KI), *Lgr6EGFPiresCreER*^{T2} (*Lgr6* KI), and *Lrig1EGFPiresCreER*^{T2} (*Lrig1* KI) mouse lines to target each stem cell compartment (Figure 1A). We collected tail skin from adult mice and analyzed epidermal EGFP expression to confirm the location of cells expressing each marker. As reported previously, *Lgr5*-EGFP$^+$ cells were found in the cycling portion of both telogen and anagen HFs (Figure 1B) (Jaks et al., 2008). *Lrig1*-EGFP$^+$ cells were mainly found in the HF junctional zone (Figure 1C) but were also found along the periphery of the SG, in the late telogen hair germ, and in the anagen hair bulb and outer root sheath, in agreement with previous publications (Jensen et al., 2009; Page et al., 2013). *Lgr6*-EGFP expression in the HF isthmus (Snippert et al., 2010), SG, and lower HF (Figure 1D) was also confirmed (Liao and Nguyen, 2014; Page et al., 2013). Significant overlap between LRIG1 and *Lgr5*-EGFP expression was found in the lower HF, particularly in

anagen follicles (Figure 1B). LRIG1 was co-expressed with *Lgr6*-EGFP at the junction between the isthmus and the junctional zone of the upper HF and the periphery of the SG (Figure 1E).

Scattered *Lgr6*-EGFP$^+$ cells were also found in tail IFE (Figure 1D). Mouse tail IFE contains two distinct lineages of keratinocytes that form the parakeratotic scales and orthokeratotic interscales (Gomez et al., 2013). Immunostaining for the interscale markers K10 and Filaggrin (FLG) (Gomez et al., 2013) established that *Lgr6*-EGFP$^+$ IFE cells were largely confined to the interscale IFE and absent from the scale IFE (Figures S1A and S1B).

For genetic lineage tracing (Kretzschmar and Watt, 2012), we bred KI mice with mice carrying the *R26R-tdTomato* reporter (Figure 1F). As a control for leakiness of the CreER constructs, some mice were treated with acetone in the absence of 4-hydroxy-tamoxifen (4-OHT) (Figure S2). In acetone-treated *Lgr5* KI/*R26R-tdTomato* mice, patches of tdTomato$^+$ cells were detected in the lower HF in the absence of 4-OHT (Figure S2A). Some leakiness was observed in hair germ, junctional zone, infundibulum, and sebaceous duct of acetone-treated *Lrig1* KI/*R26R-tdTomato* control mice (Figure S2B). Acetone-only-treated *Lgr6* KI/*R26R-tdTomato* mice showed very little activation of Cre (Figure S2C). Overall, the leakiness was minimal and specific to the expected regions of Cre expression.

When *Lgr5* KI/*R26R-tdTomato* mice were treated with one dose of 4-OHT and examined 2 weeks later, tdTomato$^+$ cells were found in the hair germ and bulge (Figure 1G), as reported previously (Jaks et al., 2008). In *Lrig1* KI/*R26R-tdTomato* mice, tdTomato$^+$ cells were present in the lower SG, HF junctional zone, and infundibulum (upper PSU), as well as in the hair germ (Figure 1H), again confirming previous observations (Page et al., 2013). In contrast, the progeny of LGR6$^+$ stem cells contributed to the upper HF, SG, and IFE (Figure 1I), confirming the results for back skin previously obtained by Snippert et al. (2010). In the tail IFE, tdTomato$^+$ progeny of *Lgr6*-EGFP$^+$ cells were largely confined to the interscale IFE, which expresses keratin 2 (K2) in addition to K10 and Filaggrin (Figure S1D) (Gomez et al., 2013), but absent from the K31$^+$ scale IFE (Figure S1E). tdTomato$^+$ progeny of *Lgr6*-expressing cells were present in all IFE layers and were readily detectable even 6 months after 4-OHT treatment (Figures S1D and S1F), suggesting that LGR6 marks interscale IFE stem cells.

These results confirm previous observations that LGR5, LGR6, and LRIG1 are markers of distinct epidermal subpopulations. In addition, they establish LGR6 as a marker to distinguish interscale from scale stem cells.

Stem Cell Origins of Ectopic Hair Follicles

To study the contribution of each stem cell population to ectopic HF formation, we bred KI mice with ΔK5ΔNβ-cateninER^t (ΔK5β-catER) transgenic mice (Baker et al., 2010) carrying the *R26R-tdTomato* reporter (Figure 2A). Adult mice were treated with one dose of 4-OHT to simultaneously induce Cre and ectopic HF formation (Figure 2B). Tissue was collected 1 or 2 weeks following 4-OHT application, by which time ectopic HF formation was evident (Figures 2B and S3B). Activation of the ΔK5β-catER transgene stimulated anagen of existing HFs and induced conversion of SGs into ectopic HFs (Figure S3). Cells in ectopic HFs and the base of anagen HFs expressed CDP, an inner root sheath/hair bulb marker, as reported previously (Figures 2C–2E) (Baker et al., 2010; Lo Celso et al., 2004). CDP expression was not detected in SGs of 4-OHT-treated mice lacking the ΔK5β-catER transgene, but CDP was expressed in some cells of the hair germ (Figure S4A).

In 4-OHT-treated *Lgr5* KI/*R26R-tdTomato*/ΔK5β-catER mice, robust tdTomato labeling was detected in the lower hair bulge and early anagen bulbs (Figure 2C). tdTomato$^+$ progeny of *Lgr5*-expressing cells were present in ~11% of ectopic HFs (Figure 2F), where they were confined to the inner cell layers (Figure S4B). Some EGFP$^+$ cells were detected in ectopic HFs, consistent with *Lgr5* being a Wnt target gene (Figure 2G).

Over 90% of ectopic HFs in *Lrig1* KI/*R26R-tdTomato*/ΔK5β-catER epidermis contained clusters of tdTomato$^+$ cells (Figures 2D and 2F). Ectopic HFs were also strongly positive for *Lrig1*-EGFP (Figure 2G). Almost 100% of ectopic HFs arising from SGs in *Lgr6* KI/*R26R-tdTomato*/ΔK5β-catER mice were strongly labeled with tdTomato (Figures 2E and 2F), and *Lgr6*-EGFP$^+$ cells were found in the ectopic HFs arising from the SG (Figure 2G). *Lgr6*-EGFP and LRIG1 were both expressed in ectopic HFs arising in the IFE (Figure 2I).

Although in ΔK5β-catER transgenic mice ectopic HFs arise predominantly from the SG, some ectopic HFs also form in the tail IFE (Baker et al., 2010), even after a single dose of 4-OHT (Figure 2H). The IFE ectopic HFs were derived from LGR6$^+$, but not from LRIG1$^+$ or LGR5$^+$ stem cells of the HF (Figure 2H; data not shown). tdTomato labeling in IFE ectopic HFs was not continuous with the PSU (Figure 2H), which supports the concept that the ectopic growths were of IFE origin, as previously reported (Silva-Vargas et al., 2005).

Collectively, these data demonstrate that ectopic HFs arising from the SG are not derived from *Lgr5*-expressing stem cells but originate from stem cell pools expressing *Lrig1* and *Lgr6* that reside at, or in close proximity to, the sites of ectopic HF formation (Figure 2J). Ectopic HFs often comprised a mixture of tdTomato-positive and negative cells (Figures 2E–2G), indicating a polyclonal origin. LGR6$^+$ IFE cells contributed to ectopic HFs arising from the IFE, whereas LRIG1$^+$ and LGR5$^+$ HF cells did not (Figure 2J).

β-Catenin Activation in Different Stem Cell Populations Results in Different Tumor Types

To study the contribution of different stem cell populations to β-catenin-induced epidermal tumors, we generated KI/*R26R-tdTomato*/*Ctnnb1*$^{lox(ex3)/+}$ mice and treated adult animals with a single dose of 4-OHT. Cre-dependent deletion of exon 3 of *Ctnnb1* (*Ctnnb1*$^{lox(ex3)}$) results in constitutive activation of β-catenin in the respective stem cell populations and their progeny (Harada et al., 1999) (Figures 3A and 3B). Control mice that lacked the *Ctnnb1* mutant allele did not exhibit any abnormal phenotypes (Figures 3C–3F).

Lgr5 KI/*R26R-tdTomato*/*Ctnnb1*$^{lox(ex3)/+}$ mice entered anagen 2 weeks following 4-OHT application, and pilomatricomas, benign HF skin tumors (Chan et al., 1999; Lo Celso et al., 2004; Merrill et al., 2001), developed within 4 weeks (Figures 3A–3I). The upper portions of the HF, SGs, and IFE were unaffected by the lesions arising in the lower HF (Figures 3D–3M).

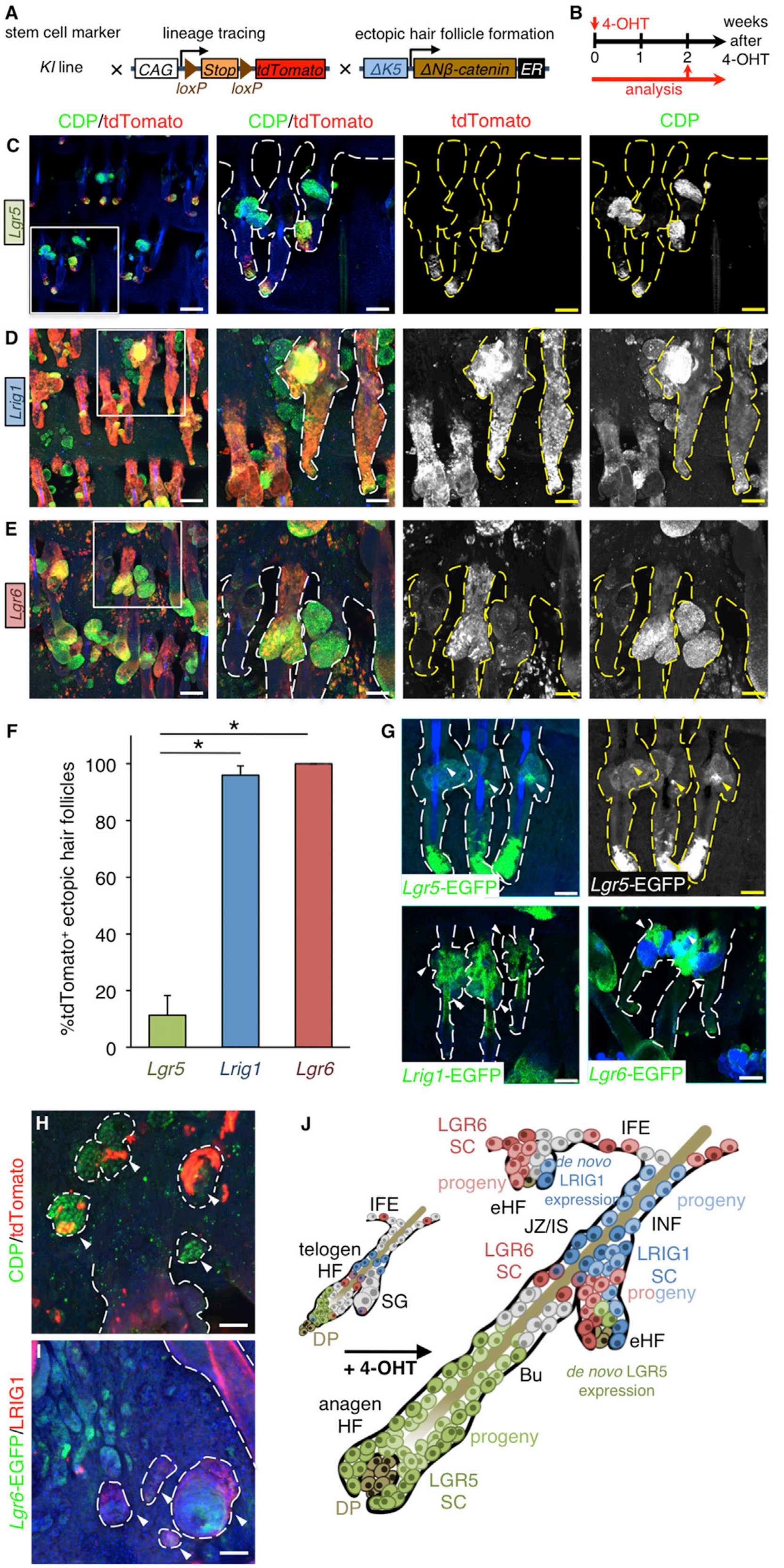

Figure 2. Lineage Tracing of Ectopic Hair Follicles

(A and B) Schematic representation of the genetic elements for lineage tracing (A) and experimental setup (B).

(C–E and G–I) Tail epidermal whole mounts collected 2 weeks after 4-OHT treatment and stained with anti-tdTomato, anti-CDP, and anti-GFP, as indicated. Boxed regions in (C)–(E) are also shown at higher magnification. (C and G) *Lgr5* KI/*R26R-tdTomato*/ΔK5ΔNβ-cateninER; (D and G) *Lrig1* KI/*R26R-tdTomato*/ΔK5ΔNβ-cateninER; (E, G, H, and I) *Lgr6* KI/*R26R-tdTomato*/ΔK5ΔNβ-cateninER. Whole mounts were counterstained with DAPI to label nuclei or with phalloidin to label F-actin (blue). Single-color images for some markers are shown in grayscale. Dashed lines demarcate HF, SG and ectopic HFs. Individual ectopic HFs are indicated with arrowheads.

(F) % pilosebaceous units (PSUs) with tdTomato$^+$ ectopic HFs (10–25 PSUs per mouse, n = 3). Data are mean ± SEM. *p < 0.05.

(J) Schematic showing the cellular origins of ectopic HF (eHF).

Bu, bulge; DP, dermal papilla; HG, hair germ; IFE, interfollicular epidermis; JZ/IS, junctional zone/isthmus; INF, infundibulum; SC, stem cell. Scale bars represent 100 μm (C–E and G) and 50 μm (H and I).

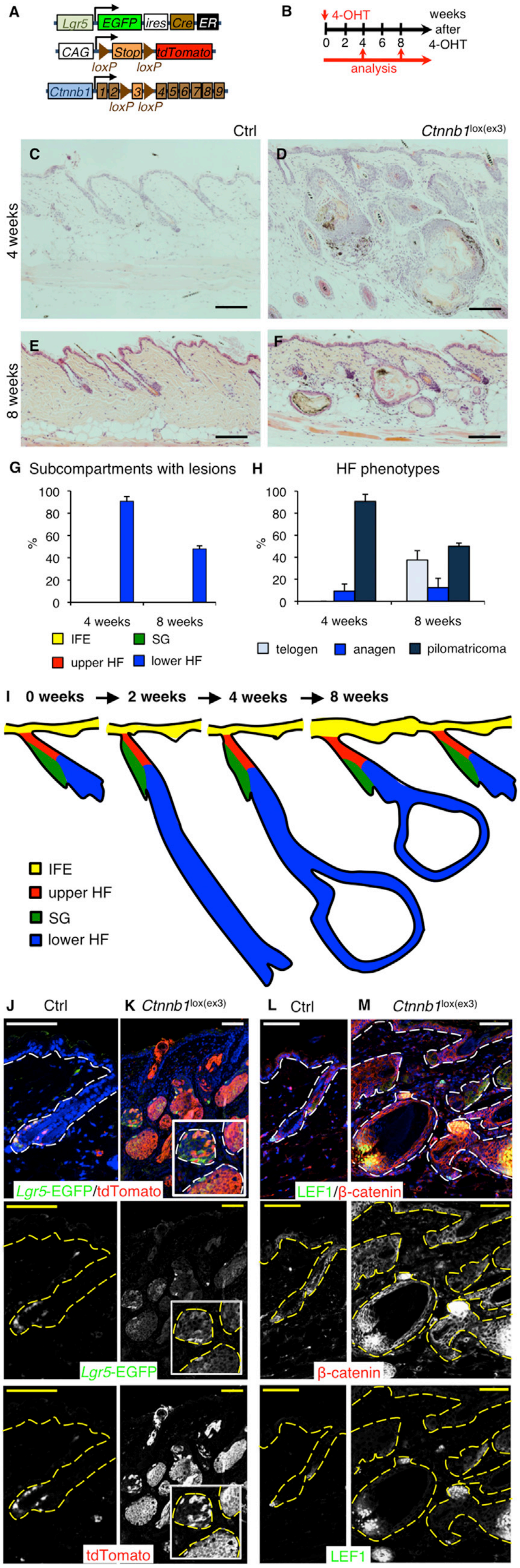

Figure 3. Oncogenic Activation of β-Catenin in LGR5$^+$ Stem Cells

(A) Schematic representation of the genetic elements for lineage tracing and β-catenin activation.

(B) Experimental setup.

(C–F and J–M) Back skin sections of *Lgr5* KI/*R26R-tdTomato*/*Ctnnb1*$^{lox(ex3)/+}$ mutant and control (Ctrl) mice 4 (C, D, J–M) and 8 weeks after 4-OHT treatment (E and F). (C–F) H&E staining. (J–M) Immunolabeled with the antibodies indicated and counterstained with DAPI to label nuclei (blue). Grayscale images of the same fields are also shown. Dashed lines denote epidermal-dermal boundaries. Insert shows higher magnification view of selected area. Scale bars, 200 μm.

(G and H) Quantification of lesions in all epidermal subcompartments (G) and lower HF phenotypes (H). Data are means ± SD (25 PSUs scored per mouse; 4 weeks, n = 3; 8 weeks, n = 2).

(I) Schematic of phenotype progression over time. Scale bars, 200 μm.

Eight weeks following 4-OHT application, most HFs had re-entered telogen, but pilomatricomas persisted, extending into the skin adipocyte layer (Figures 3F, 3H, and 3I; 50 PSUs scored from two mice). Mice carrying the mutant allele of *Ctnnb1* had to be killed 8 weeks following 4-OHT induction due to weight loss. The mice had developed small intestinal tumors most likely caused by 4-OHT ingestion activating the mutant allele in *Lgr5*-expressing cells of the gastrointestinal (GI) tract (data not shown) (Barker et al., 2009).

In *Lgr5* KI/*R26R-tdTomato*/*Ctnnb1*$^{lox(ex3)/+}$ mutant and *Lgr5* KI/*R26R-tdTomato*/*Ctnnb1*$^{+/+}$ control skin tdTomato$^+$ cells were only present in the lower HF (bulge and hair germ) and were absent from the permanent portion of the HF (Figures 3J and 3K). No *Lgr5*-EGFP$^+$ or tdTomato$^+$ cells were found in the dermis (Figures 3J and 3K). Immunostaining for β-catenin and LEF1 indicated that Wnt signaling was restricted to anagen bulbs and HF tumors (Figures 3L and 3M; data not shown). HF tumors were strongly positive for the proliferation marker Ki67 (Figure S5A) and expressed the HF differentiation markers K17 and K31 (Figures S5B and S5C).

In contrast to *Lgr5* KI/*R26R-tdTomato*/*Ctnnb1*$^{lox(ex3)/+}$ mice, adult *Lrig1* KI/*R26R-tdTomato*/*Ctnnb1*$^{lox(ex3)/+}$ mice (Figures 4A and 4B) developed a hyperplastic, highly keratinized junctional zone and a thickening of the IFE within 4 weeks following 4-OHT application (Figures 4C, 4F, and 4G). The phenotype became progressively more severe over the subsequent 6 weeks after 4-OHT treatment, affecting the entire HF (Figures 4D–4G). The lesions resembled human trichoadenomas, a rare benign follicular tumor with cornifying cysts (Kurokawa et al., 2005; Shimanovich et al., 2010), with a high proportion of undifferentiated cells and an infiltrate of immune cells including neutrophils (Figure S6A). *Lrig1* KI/*R26R-tdTomato*/*Ctnnb1*$^{lox(ex3)/+}$ mice had to be killed within 6 weeks of the start of the experiment, as they developed GI tumors, consistent with *Lrig1* expression in the intestinal epithelium (Powell et al., 2012; Wong et al., 2012).

Lineage tracing revealed that at 4 weeks tdTomato$^+$ cells were mostly restricted to the tumors, the upper portion of the HF and IFE (Figures 4H and 4I). In agreement with previous publications, cells positive for tdTomato and *Lrig1*-EGFP were also found in the dermis (Driskell et al., 2013; Gomez et al., 2013) (Figures 4H and 4I). β-Catenin and LEF1 expression was highly upregulated in the hyperplastic HF junctional zone and tumors of *Ctnnb1* mutant skin (Figure 4J). However, nuclear β-catenin

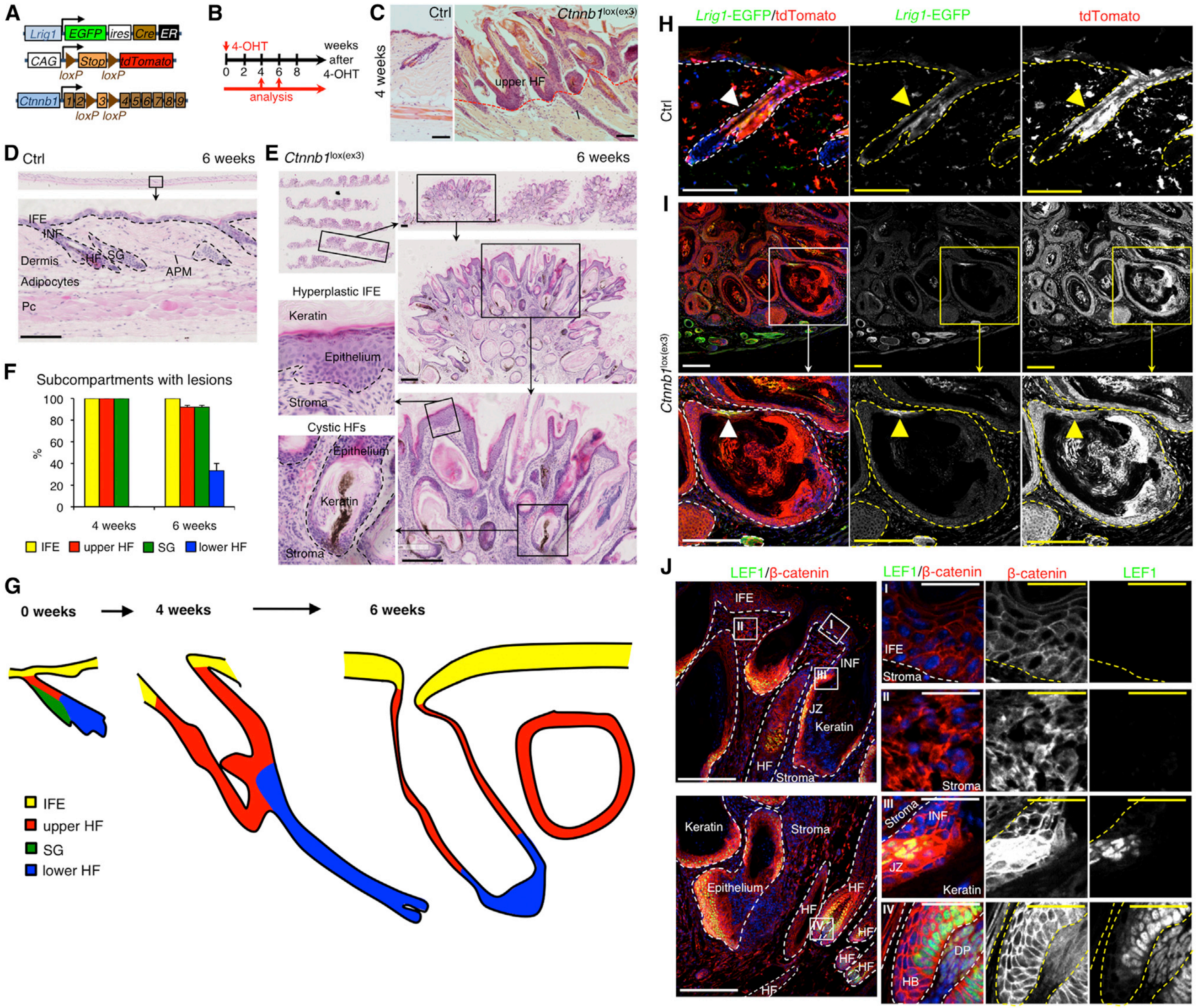

Figure 4. Oncogenic Activation of β-Catenin in LRIG1$^+$ Stem Cells

(A) Schematic representation of genetic elements for lineage tracing and β-catenin activation.

(B) Experimental setup.

(C–E and H–J) Back skin sections of *Lrig1* KI/*R26R-tdTomato*/*Ctnnb1*$^{lox(ex3)/+}$ mutant and control (Ctrl) mice 4 weeks (unless stated otherwise) after 4-OHT application, stained with H&E (C–E) or antibodies to the markers indicated, with DAPI counterstain to label nuclei (blue) (H–J). Grayscale images for the same fields are also shown (H–J). Dashed lines and arrowheads denote epidermal-dermal boundaries. Inserts show higher magnification views of selected areas. Scale bars represent 200 μm, except (J.I)–(J.IV) (50 μm).

(F) Quantification of lesions in all dorsal epidermal subcompartments with abnormalities. 25 PSUs per mouse (n = 3). Data are means ± SEM.

(G) Schematic of phenotype progression over time.

and LEF1 were not detected in the IFE or tumor stroma, suggesting a lack of significant Wnt/β-catenin activity in these compartments (Figure 4J).

Expression of K14 extended into the suprabasal layers of back skin IFE and cysts, while expression of the differentiation markers K10 and involucrin (IVL) was perturbed in the suprabasal layers (Figures S6B and S6C), indicating abnormal terminal differentiation, as observed in human trichoadenoma (Kurokawa et al., 2005). Pronounced expression of cyclin D1 and Ki67 in the trichoadenoma cysts (Figures S6D and S6E) confirmed hyperplasia, again consistent with the human lesions (Kurokawa et al., 2005; Shimanovich et al., 2010).

Within 4 weeks of 4-OHT treatment (Figures 5A and 5B), *Lgr6* KI/*R26R-tdTomato*/*Ctnnb1*$^{lox(ex3)/+}$ mutant skin developed ectopic HFs from the SG and IFE and exhibited expansion of the upper portion of the HF (Figures 5C, 5D, 5H–5J, and 5L). By 12 weeks, cysts had formed in the SG and upper and lower HF (Figures 5F–5L). The IFE of *Lgr6* mutant skin progressively developed irregular invaginations into the underlying dermis resembling the epithelial compartment of a benign human skin

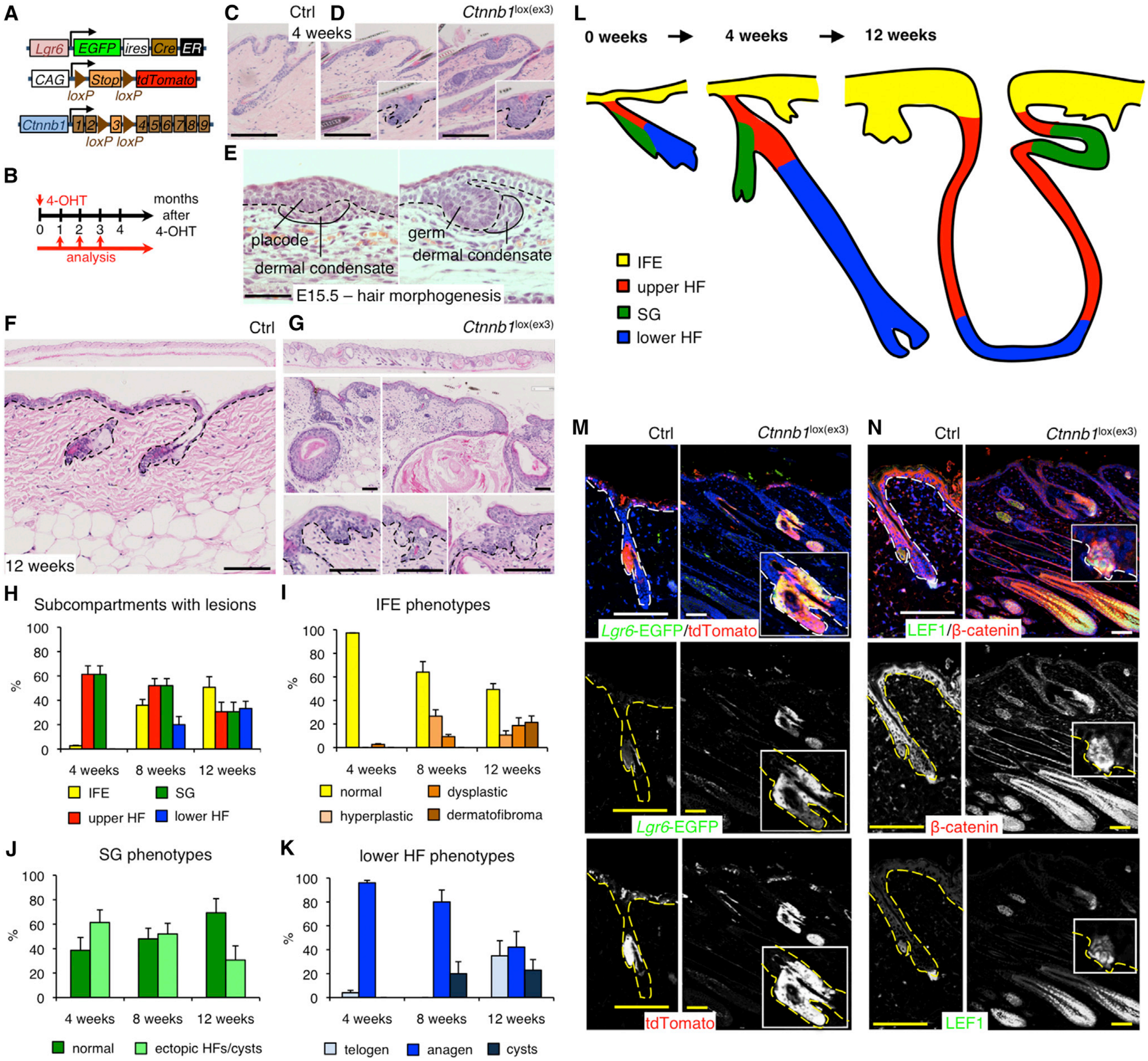

Figure 5. Oncogenic Activation of β-Catenin in LGR6$^+$ Stem Cells

(A) Schematic representation of genetic elements for lineage tracing and oncogenic β-catenin activation in LGR6$^+$ stem cells.

(B) Experimental setup.

(C, D, F, G, M, and N) Back skin sections of *Lgr6* KI/*R26R-tdTomato*/*Ctnnb1*$^{lox(ex3)/+}$ mutant and control (Ctrl) mice 4 weeks (unless stated otherwise) after 4-OHT application, stained with hematoxylin and eosin (H&E; C, D, F, and G) or antibodies to the markers indicated, with DAPI counterstain to label nuclei (blue) (M and N). Grayscale images for the same fields are also shown. Dashed lines denote epidermal-dermal boundaries. Inserts show higher magnification views of selected areas.

(E) E15.5 sections of wild-type embryonic skin stained with hematoxylin and eosin.

(H–K) Quantification of lesions in all dorsal epidermal subcompartments with abnormalities (H) and specific abnormalities in IFE (I), SG (J), and HF (K). 25 PSUs per mouse (n = 3). Data are means ± SEM.

(L) Schematic of phenotype progression over time.

Scale bars represent 200 μm, except (E) (100 μm).

lesion called dermatofibroma (or benign fibrous histiocytoma) (Figures 5G, 5I, and 5L). Dermatofibroma is characterized by pronounced epidermal hyperplasia and occasional HF budding that mimics superficial BCCs (Zelger et al., 2004). At four weeks, the HF outgrowths in mouse skin resembled embryonic hair placodes and hair germs (Figures 5D and 5E). Some developed

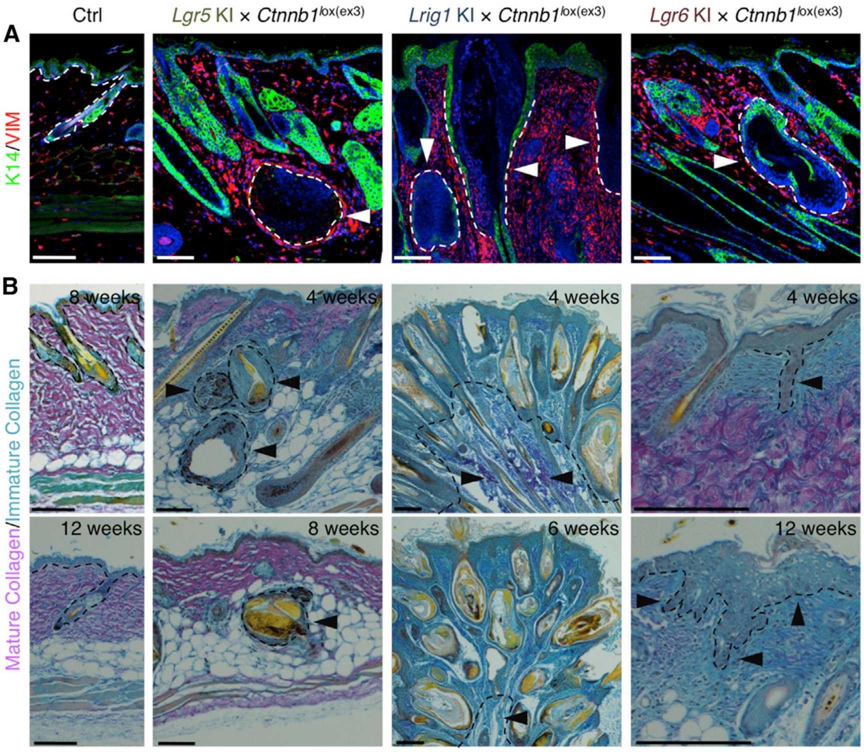

Figure 6. Differential Stromal Responses to Compartmentalized Epidermal Activation of β-Catenin

(A and B) Back skin sections of *Ctnnb1*$^{lox(ex3)/+}$ mutant and control (Ctrl) mice indicated, 4 weeks (unless stated otherwise) after 4-OHT application. (A) Stained with anti-K14 (green) and anti-vimentin (VIM; red), with DAPI counterstain (blue). (B) Stained with Herovici dye to visualize immature collagen (light blue) and mature collagen (pink). Dashed lines and arrowheads denote epithelial-stromal boundaries. Scale bars, 200 μm.

into small HFs with associated SGs (Figure S7A) but progressed into disorganized lesions (Figure 5G).

Expression of *Lgr6*-EGFP and tdTomato was compartmentalized in *Ctnnb1* mutant skin, with cells positive for either or both markers being present in the IFE and hyperplastic HF junctional zone as well as ectopic HFs arising from the SG (Figure 5M). In *Ctnnb1* mutant skin, β-catenin, LEF1, and cyclin D1 expression was highly upregulated in cells in tumors and ectopic HFs (Figures 5N and S7B). IFE dysplasias were also strongly positive for the proliferation marker Ki67 and showed defects in differentiation (Figures S7C and S7D). Infundibular cysts and HF buds arising from the IFE were positive for the HF lineage marker K17 (Figure S7E).

These experiments show that sustained activation of β-catenin in different stem cell compartments results in tumors that differ in location and type.

Distinct Dermal Responses to Compartmentalized Stabilization of Epidermal β-Catenin

Sustained activation of β-catenin throughout the basal layer of adult epidermis reprograms the underlying dermis to a neonatal state, characterized by an increase in fibroblast density and extensive extracellular matrix (ECM) remodeling (Collins et al., 2011). To discover whether, in addition to inducing different types of tumors, β-catenin activation in different stem cell subpopulations had different effects on the dermis, we first evaluated skin for fibroblast density by labeling for vimentin and for ECM remodeling by labeling for CD44 and staining with Herovici. CD44 is a β-catenin target gene (Wielenga et al., 1999) and the major cell-surface receptor for hyaluronic acid (hyaluronan), a key ECM component (Edward et al., 2005). Herovici is a histochemical dye that stains highly crosslinked, mature collagen fibers pink and stains fine, immature collagen fibrils light blue (Collins et al., 2011).

Pilomatricomas resulting from *Ctnnb1* activation in *Lgr5*-expressing stem cells were surrounded by an increased density of vimentin^{+} fibroblasts, while fibroblast density in the rest of the skin was unaffected (Figure 6A). Conversely, when stabilized β-catenin was expressed using the *Lrig1* KI and *Lgr6* KI mouse models, only the upper layers of the dermis exhibited an increased density of fibroblasts (Figure 6A).

At 4 weeks, back skin of *Lgr5* KI/*R26R-tdTomato*/*Ctnnb1*$^{lox(ex3)/+}$ mutant mice showed comprehensive dermal ECM remodeling and CD44 expression in the lower dermis surrounding the pilomatricomas, while the upper dermis contained only mature collagen (Figures 6B and S5D). By 8 weeks, the entire dermis contained mature collagen (stained in pink), even though the tumors persisted (Figure 6B).

Activation of the *Ctnnb1* mutant using the *Lrig1* KI and *Lgr6* KI mouse models stimulated ECM remodeling initially only around hyperplastic sites (Figure 6B). As the tumor lesions progressed in the back skin of 4-OHT-treated *Lrig1* KI/*R26R-tdTomato*/*Ctnnb1*$^{lox(ex3)/+}$ mice, collagen was comprehensively remodeled and the dermis was almost entirely filled with compact immature collagen characteristic of tumor stroma (Figure 6B). CD44 expression in fibroblasts was confined to the areas surrounding the tumors but was also highly upregulated in the hyperplastic epidermal regions of the tumor itself (Figure S6F). In *Lgr6* KI/*R26R-tdTomato*/*Ctnnb1*$^{lox(ex3)/+}$ skin, the dermis underlying IFE dysplasias and surrounding upper pilosebaceous cysts stained light blue with Herovici, indicating the presence of immature collagen fibrils (Figure 6B). By 8 weeks, almost the entire dermal ECM contained immature collagen (Figure 6B). Again, CD44 expression was selectively upregulated in the fibroblasts adjacent to tumors (Figure S7F).

We also examined dermal expression of CD45 and CD26. CD45 is expressed by infiltrating immune cells (Arwert et al., 2012a; Leibl et al., 1985). CD26, a T cell activation antigen also

OPEN
ACCESS
CellPress

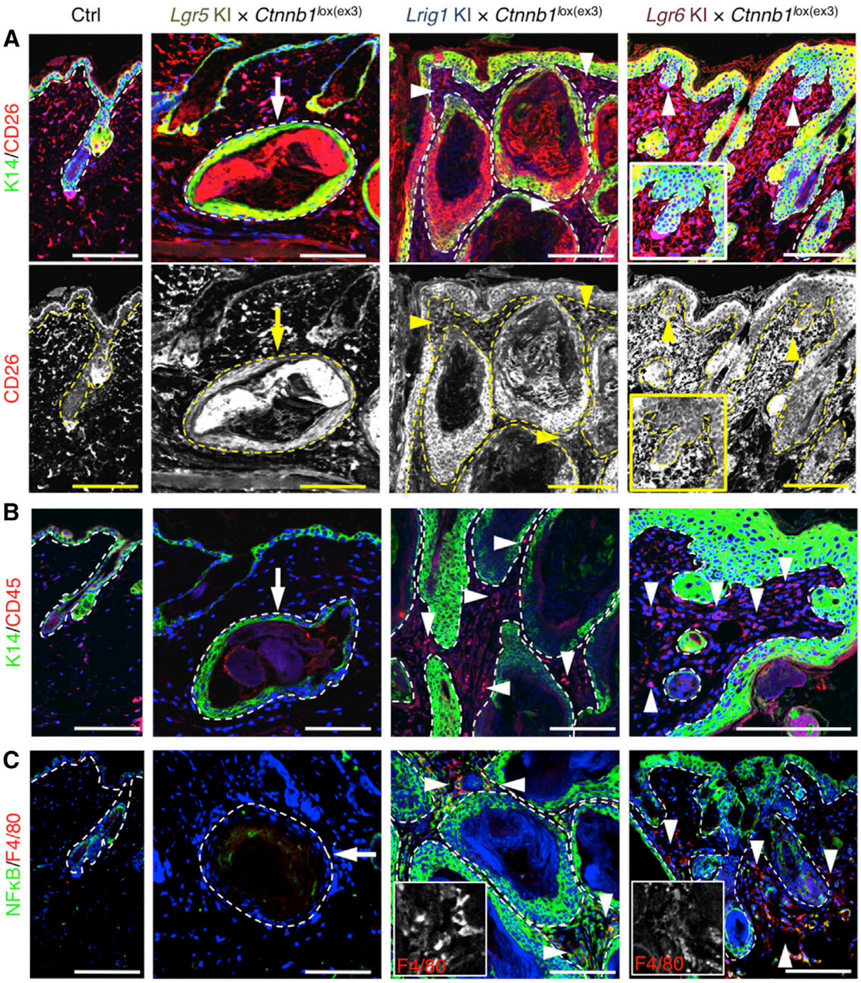

Figure 7. Inflammatory Response to Compartmentalized Epidermal β-Catenin Activation

(A–C) Back skin sections collected from *Ctnnb1*$^{lox(ex3)/+}$ mutant and representative control (Ctrl) mice 12 (*Lgr6* KI), 8 (*Lgr5* KI), and 6 weeks (*Lrig1* KI) after 4-OHT application, stained with antibodies to the markers indicated and counter-stained with DAPI (blue). Grayscale images show the same field (A) or higher-magnification views of selected areas (C). Arrows show lack of staining, and arrowheads show positive staining in the stroma. Dashed lines indicate boundary between epithelium and stroma. Scale bars, 200 μm.

known as dipeptidyl peptidase-4 (DPP4), is upregulated in hyperproliferative epidermis and in dermal fibroblasts during wounding and chronic inflammation but is downregulated in the stroma of epidermal tumors (Arwert et al., 2012b; Morimoto and Schlossman, 1998).

In mice carrying the *Lgr5* KI and *Ctnnb1* mutant alleles, no differences in expression of CD26 and CD45 were found at any time up to 8 weeks following 4-OHT treatment (Figures 7A and 7B). When the *Ctnnb1* activating mutation was induced in LRIG1$^+$ stem cells, CD26 was highly upregulated in epidermal keratinocytes but was reduced in fibroblasts surrounding the tumors (Figure 7A). CD45$^+$ cells were present in the dermis adjacent to the epidermal cysts and underlying the IFE (Figure 7B). β-Catenin stabilization in *Lgr6*-expressing stem cells in the upper epidermis stimulated dermal CD26 expression (Figure 7A) and resulted in an increase in CD45$^+$ cells (Figure 7B).

To further investigate the inflammatory response (Arwert et al., 2012a; Hanahan and Weinberg, 2011) to epidermal β-catenin activation, we examined expression of the transcription factor nuclear factor kappa-light-chain-enhancer of activated B cells (NF-κB) and the macrophage marker F4/80. NF-κB signaling and subsequent recruitment of immune cells creates a pro-tumorigenic microenvironment in skin (Arwert et al., 2010; Karin, 2009).

In control and *Lgr5* KI/*R26R-tdTomato*/*Ctnnb1*$^{lox(ex3)/+}$ mutant skin, NF-κB and F4/80 were not detectable in the dermis (Figure 7C). In contrast, skin from mice carrying the *Ctnnb1* mutant allele with the *Lgr6* KI or *Lrig1* KI had strongly elevated expression of NF-κB in both epidermis and dermis (Figure 7C). F4/80$^+$ macrophages were present in regions of the tumor stroma with high NF-κB expression (Figure 7C).

In summary, the compartmentalized induction of epidermal β-catenin triggered different dermal responses, reflecting differences in the behavior of fibroblasts and immune cells. HF tumors in *Lgr5* KI/*R26R-tdTomato*/*Ctnnb1*$^{lox(ex3)/+}$ mutant skin arose in the absence of inflammation, while tumors initiated in the permanent portion of the epidermis of *Lgr6* KI/*R26R-tdTomato*/*Ctnnb1*$^{lox(ex3)/+}$ or *Lrig1* KI/*R26R-tdTomato*/*Ctnnb1*$^{lox(ex3)/+}$ mice were characterized by stromal inflammation (Arwert et al., 2012a; Hanahan and Weinberg, 2011).

DISCUSSION

Here, we show that different epidermal stem cell populations exhibit different responses to transient or sustained activation of Wnt/β-catenin. We identify the cells of origin of ectopic HFs and three different types of epidermal tumor. We show that different tumor types have a distinct stromal signature, and we identify the stem cell compartment that gives rise to the orthokeratotic interscale IFE in the tail.

By targeting distinct epidermal stem cell compartments in the lower (hair germ and bulge) and upper PSU (HF isthmus, junctional zone, infundibulum, and SG) and IFE, we traced the cellular origin of ectopic HFs induced by transient epidermal β-catenin activation in the SG and orthokeratotic IFE. Ectopic HFs did not originate from LGR5$^+$ stem cells in the lower HF, even though

OPEN
ACCESS
CellPress

they are β-catenin sensitive (Jaks et al., 2008), and LGR5$^+$ cells in the new HFs arose from LGR5$^-$ cells, suggesting that ectopic HFs indeed recapitulate the process of HF development, which has been proposed previously (Baker et al., 2010; Lo Celso et al., 2004; Silva-Vargas et al., 2005). Thus, β-catenin-sensitive LGR5$^+$ cells in the lower HF were not redirected to sites of ectopic HF formation in the epidermal compartments above the bulge.

In contrast, the progeny of LGR6$^+$ and LRIG1$^+$ epidermal stem cells robustly contributed to ectopic HFs arising from the SG. None of the new follicles were 100% tdTomato$^+$, demonstrating that they were polyclonal in origin, as reported previously (Silva-Vargas et al., 2005). We conclude that β-catenin activation within the SG causes a direct lineage switch of stem cell progeny from sebocytes to HF lineages, and tissue compartmentalization is maintained (Füllgrabe et al., 2015; Page et al., 2013).

The progeny of LGR6$^+$ stem cells have previously been traced into the IFE, SG, and upper HF, suggesting that a single pool of *Lgr6*-expressing stem cells renews the entire permanent portion of adult epidermis (Snippert et al., 2010). However, two subsequent studies identified scattered *Lgr6*-EGFP$^+$ cells in multiple compartments of adult mouse back skin, including the lower HF, SG, and IFE (Figure 1D) (Liao and Nguyen, 2014; Page et al., 2013), and a recent study has demonstrated that LGR6$^+$ cells in all these regions contribute to epidermal homeostasis (Füllgrabe et al., 2015). We were able to show that ectopic HFs arising from the IFE are derived from *Lgr6*-expressing cells in the interscale of tail IFE (Figures 2H and 2I), strongly suggesting that LGR6$^+$ cells constitute the interscale stem cell pool (Gomez et al., 2013). In contrast, the scale IFE of tail skin did not express *Lgr6* (Figure S1). Some of the hair placodes originating from LGR6$^+$ IFE matured into hair-shaft-producing HFs that had associated SGs, upon activation of the *Ctnnb1*$^{lox(ex3)}$ mutant allele (Figure S7A), establishing the competence of the IFE to undergo HF neogenesis in adult murine back skin.

Tumor formation was readily initiated from all the epidermal compartments in back skin upon genetic stabilization of β-catenin. Further triggers such wounding or 7,12-dimethylbenz[α]anthracene (DMBA)/12-O-tetradecanoylphorbol 13-acetate (TPA) treatment, as reported for expression of oncogenic K-Ras (Page et al., 2013), were not required. Compartmentalization was maintained, such that tumors arising from LGR5$^+$ cells in the lower HF did not involve the upper HF or IFE, and tumors of LRIG1$^+$ and LGR6$^+$ cells in the upper HF spared the lower HF. Only in the very late stages of tumor progression was tissue integrity destroyed as a result of expansion of the tumor mass.

Deregulated Wnt signaling in skin has previously been associated with different types of tumors (Klaus and Birchmeier, 2008; Watt and Collins, 2008). We now show that tumor type depends, at least in part, on the stem cell compartment in which β-catenin is activated. LGR5$^+$ cells gave rise to pilomatricomas, while LRIG1$^+$ cells formed trichoadenomas and LGR6$^+$ cells formed infundibular cysts in the HF junctional zone and dermatofibromas in the IFE. Although dermatofibromas are characterized by increased fibroblast density and comprehensive ECM remodeling in the reticular dermis, it has been unclear whether these changes reflect oncogenic changes in the fibroblasts themselves or a response to changes in the epidermis (Calonje, 2001; Chen et al., 2000). Our study implicates the epidermis as the oncogenic target in dermatofibromas. Of note, studies have shown that some dermatofibromas resemble superficial BCCs (Zelger et al., 2004), and the β-catenin-induced dermatofibromas in our study expressed typical BCC markers such as K17 and cyclin D1 (Youssef et al., 2012). It has previously been suggested that BCCs can arise from IFE cells in response to activating mutations in the Hedgehog (Hh) signaling pathway (Grachtchouk et al., 2011; Youssef et al., 2010, 2012). Studies have also shown that β-catenin activation is required for progression of BCC in human and BCC-like lesions in mice (Pálmer et al., 2008; Youssef et al., 2012).

The oncogenic effects of β-catenin activation in LGR6$^+$ stem cell subpopulations not only caused different phenotypes depending on their location (IFE, dermatofibromas; HF junctional zone, infundibular cysts) but also took longer to develop in the IFE than the HF junctional zone, as recently reported for Hh-induced BCCs (Peterson et al., 2015). It has been suggested that the IFE is resistant to Wnt/β-catenin signaling (Watt and Collins, 2008), since epidermal deletion of β-catenin primarily affects the HFs and SGs (Huelsken et al., 2001) and ectopic HFs only arise in the IFE following strong β-catenin activation (Lo Celso et al., 2004; Silva-Vargas et al., 2005). However, there is recent evidence that Wnt signaling does control IFE stem cell proliferation, but its activity is tightly regulated to control the balance between self-renewal and differentiation of IFE stem cells (Choi et al., 2013; Lim et al., 2013). This might explain the slow development of neoplastic lesions in the IFE of *Lgr6* KI mice carrying the *Ctnnb1* activating mutation.

It has previously been shown that epidermal activation of β-catenin throughout the epidermal stem cell compartment leads to reprogramming of the dermis to a neonatal state characterized by increased fibroblast density and extensive ECM remodeling (Collins et al., 2011). The fibroblast lineages of the upper and lower dermis are responsive to epidermal β-catenin activity (Driskell et al., 2013), as is the hypodermal adipocyte layer (Donati et al., 2014). Here, we show that genetic stabilization of β-catenin in different epidermal stem cell populations resulted in compartmentalized dermal responses. In each case, there was a local increase in fibroblast density and transient ECM remodeling.

Different stromal responses to β-catenin activation in the different stem cell compartments led to distinct stromal signatures. The influx of inflammatory cells elicited by the LRIG1$^+$ compartment could reflect the expansion of the infundibulum and associated defects in the epidermal barrier (Chiang et al., 2013) or activation of β-catenin in LRIG1$^+$ fibroblasts (Gomez et al., 2013). The different tumor types also had different effects on CD26 expression: CD26 expression was unchanged when LGR5$^+$ cells were targeted, was upregulated on targeting of LGR6$^+$ cells, and was downregulated by targeting LRIG1$^+$ cells. During skin development, CD26$^+$ fibroblasts reside in the papillary dermis, which provides a permissive microenvironment for HF induction (Driskell et al., 2013). Upregulation of CD26 also occurs in response to epidermal IL1α and wounding but is downregulated in the stroma of papillomas and SCCs (Arwert et al., 2012b). It remains to be determined whether the upregulation of CD26 reflects a selective expansion of the upper dermal lineage.

In summary, we have shown that epidermal compartmentalization is sustained during activation of epidermal Wnt/β-catenin signaling in adult skin. Stem cells differ in their contribution to ectopic HFs, and stem cell heterogeneity leads to formation of different tumor types with different stromal composition. Our study highlights the functional significance of the different epidermal stem cell populations.

EXPERIMENTAL PROCEDURES

Mice

All animal experiments were subject to institutional ethical review and performed under the terms of a UK Home Office license. *Ctnnb1*$^{lox(ex3)/+}$, ΔK5ΔNβ-cateninER^{t}, *K19CreER*^{T}, *Lgr5EGFPiresCreER*^{T2}, *Lgr6EGFPiresCreER*^{T2}, *Lrig5EGFPiresCreER*^{T2}, and *R26R-tdTomato* (Ai9 line) mice have been described previously (Baker et al., 2010; Barker et al., 2007; Harada et al., 1999; Madisen et al., 2010; Means et al., 2008; Page et al., 2013; Snippert et al., 2010). For each condition and time point, three to five mice were treated and analyzed, unless stated otherwise. Treatment regimes are described in the Supplemental Experimental Procedures.

Histology and Immunohistochemistry

Back and tail skin was fixed overnight in 4% paraformaldehyde (Sigma) and embedded in paraffin wax. Immunohistochemistry and tail epidermal whole-mount preparation were performed as described previously (Braun et al., 2003; Collins et al., 2011; Kretzschmar et al., 2015) and are described in the Supplemental Experimental Procedures.

Quantification of tdTomato-Positive Ectopic HFs

10–25 pilosebaceous units per tail epidermal whole-mount collected from each mouse were examined for tdTomato^{+} ectopic HFs arising from the SG 2 weeks after 4-OHT application. For each mouse the proportion of pilosebaceous units containing tdTomato^{+} cells was calculated. This proportion was used to calculate the mean average ± SEM from three mice per condition. Statistical analysis was performed using the unpaired Student's t test. p values of less than 0.05 were considered statistically significant.

Quantification of Abnormal Phenotypes

Scanned H&E-stained sections of 25 back skin pilosebaceous units per mouse were examined using Aperio ImageScope software (Leica) for abnormalities in epidermal subcompartments (IFE, SG, upper HF: infundibulum, junctional zone and isthmus; lower HF: bulge and hair germ), as described previously (Youssef et al., 2010). IFE phenotypes were classified as normal, hyperplastic (increased numbers of cells), dysplastic (abnormal cell morphology) (Youssef et al., 2010), or dermatofibroma-positive. SG were scored as normal or abnormal (ectopic HFs and cysts). HF phenotypes were scored in the lower HF (bulge and hair germ) as normal (anagen versus telogen) following standard histological guidelines (Müller-Röver et al., 2001), cystic (absence of intact hair shaft), or pilomatricoma positive. The proportion of lesions for each condition and the proportions of the different types of lesions per epidermal subcompartment were calculated from each mouse. This proportion was used to calculate the mean average ± SEM from three to five mice per condition and time point, unless stated otherwise.

SUPPLEMENTAL INFORMATION

Supplemental Information includes Supplemental Experimental Procedures and seven figures and can be found with this article online at http://dx.doi.org/10.1016/j.celrep.2015.12.041.

ACKNOWLEDGMENTS

We are grateful to H. Clevers and R. Toftgård for providing the *Lgr5* KI and *Lgr6* KI mouse lines, M.M. Taketo for providing the *Ctnnb1*$^{lox(ex3)/+}$ mutant mice, and K.B. Jensen for generating the *Lrig1* KI mouse line. We thank all current and former Watt lab members for suggestions and advice. We acknowledge the expert technical assistance of the core facilities of the Cambridge Stem Cell Institute and King's College London, particularly those provided by the financial support from the Department of Health via the National Institute for Health Research (NIHR) comprehensive Biomedical Research Centre award to Guy's & St Thomas' NHS Foundation Trust in partnership with King's College London and King's College Hospital NHS Foundation Trust. This work was supported by the Medical Research Council (MRC; G1100073), the Wellcome Trust (096540/Z/11/Z), and the European Union Framework 7 programme (HEALING). K.K. was the recipient of an MRC Ph.D. studentship, and C.W. was the recipient of a Marie Curie Ph.D. studentship via EU-FP7 (HEALING). R.R.D. is a London Law Medal Fellow.

Received: June 24, 2014
Revised: September 26, 2015
Accepted: December 6, 2015
Published: January 7, 2016

REFERENCES

Arwert, E.N., Lal, R., Quist, S., Rosewell, I., van Rooijen, N., and Watt, F.M. (2010). Tumor formation initiated by nondividing epidermal cells via an inflammatory infiltrate. Proc. Natl. Acad. Sci. USA *107*, 19903–19908.

Arwert, E.N., Hoste, E., and Watt, F.M. (2012a). Epithelial stem cells, wound healing and cancer. Nat. Rev. Cancer *12*, 170–180.

Arwert, E.N., Mentink, R.A., Driskell, R.R., Hoste, E., Goldie, S.J., Quist, S., and Watt, F.M. (2012b). Upregulation of CD26 expression in epithelial cells and stromal cells during wound-induced skin tumour formation. Oncogene *31*, 992–1000.

Baker, C.M., Verstuyf, A., Jensen, K.B., and Watt, F.M. (2010). Differential sensitivity of epidermal cell subpopulations to beta-catenin-induced ectopic hair follicle formation. Dev. Biol. *343*, 40–50.

Barker, N., van Es, J.H., Kuipers, J., Kujala, P., van den Born, M., Cozijnsen, M., Haegebarth, A., Korving, J., Begthel, H., Peters, P.J., and Clevers, H. (2007). Identification of stem cells in small intestine and colon by marker gene Lgr5. Nature *449*, 1003–1007.

Barker, N., Ridgway, R.A., van Es, J.H., van de Wetering, M., Begthel, H., van den Born, M., Danenberg, E., Clarke, A.R., Sansom, O.J., and Clevers, H. (2009). Crypt stem cells as the cells-of-origin of intestinal cancer. Nature *457*, 608–611.

Beronja, S., Janki, P., Heller, E., Lien, W.H., Keyes, B.E., Oshimori, N., and Fuchs, E. (2013). RNAi screens in mice identify physiological regulators of oncogenic growth. Nature *501*, 185–190.

Blanpain, C., Lowry, W.E., Geoghegan, A., Polak, L., and Fuchs, E. (2004). Self-renewal, multipotency, and the existence of two cell populations within an epithelial stem cell niche. Cell *118*, 635–648.

Blanpain, C., Horsley, V., and Fuchs, E. (2007). Epithelial stem cells: turning over new leaves. Cell *128*, 445–458.

Braun, K.M., Niemann, C., Jensen, U.B., Sundberg, J.P., Silva-Vargas, V., and Watt, F.M. (2003). Manipulation of stem cell proliferation and lineage commitment: visualisation of label-retaining cells in wholemounts of mouse epidermis. Development *130*, 5241–5255.

Brownell, I., Guevara, E., Bai, C.B., Loomis, C.A., and Joyner, A.L. (2011). Nerve-derived sonic hedgehog defines a niche for hair follicle stem cells capable of becoming epidermal stem cells. Cell Stem Cell *8*, 552–565.

Calonje, E. (2001). Dermatofibroma (fibrous histiocytoma): an inflammatory or neoplastic disorder? Histopathology *39*, 213.

Chan, E.F., Gat, U., McNiff, J.M., and Fuchs, E. (1999). A common human skin tumour is caused by activating mutations in beta-catenin. Nat. Genet. *21*, 410–413.

Chen, T.C., Kuo, T., and Chan, H.L. (2000). Dermatofibroma is a clonal proliferative disease. J. Cutan. Pathol. *27*, 36–39.

Chiang, M.F., Yang, S.Y., Lin, I.Y., Hong, J.B., Lin, S.J., Ying, H.Y., Chen, C.M., Wu, S.Y., Liu, F.T., and Lin, K.I. (2013). Inducible deletion of the Blimp-1 gene in adult epidermis causes granulocyte-dominated chronic skin inflammation in mice. Proc. Natl. Acad. Sci. USA *110*, 6476–6481.

Choi, Y.S., Zhang, Y., Xu, M., Yang, Y., Ito, M., Peng, T., Cui, Z., Nagy, A., Hadjantonakis, A.K., Lang, R.A., et al. (2013). Distinct functions for Wnt/β-catenin in hair follicle stem cell proliferation and survival and interfollicular epidermal homeostasis. Cell Stem Cell *13*, 720–733.

Collins, C.A., Kretzschmar, K., and Watt, F.M. (2011). Reprogramming adult dermis to a neonatal state through epidermal activation of β-catenin. Development *138*, 5189–5199.

Deschene, E.R., Myung, P., Rompolas, P., Zito, G., Sun, T.Y., Taketo, M.M., Saotome, I., and Greco, V. (2014). β-Catenin activation regulates tissue growth non-cell autonomously in the hair stem cell niche. Science *343*, 1353–1356.

Donati, G., Proserpio, V., Lichtenberger, B.M., Natsuga, K., Sinclair, R., Fujiwara, H., and Watt, F.M. (2014). Epidermal Wnt/β-catenin signaling regulates adipocyte differentiation via secretion of adipogenic factors. Proc. Natl. Acad. Sci. USA *111*, E1501–E1509.

Driskell, R.R., Lichtenberger, B.M., Hoste, E., Kretzschmar, K., Simons, B.D., Charalambous, M., Ferron, S.R., Herault, Y., Pavlovic, G., Ferguson-Smith, A.C., and Watt, F.M. (2013). Distinct fibroblast lineages determine dermal architecture in skin development and repair. Nature *504*, 277–281.

Edward, M., Gillan, C., Micha, D., and Tammi, R.H. (2005). Tumour regulation of fibroblast hyaluronan expression: a mechanism to facilitate tumour growth and invasion. Carcinogenesis *26*, 1215–1223.

Füllgrabe, A., Joost, S., Are, A., Jacob, T., Sivan, U., Haegebarth, A., Linnarsson, S., Simons, B.D., Clevers, H., Toftgård, R., and Kasper, M. (2015). Dynamics of Lgr6+ progenitor cells in the hair follicle, sebaceous gland, and interfollicular epidermis. Stem Cell Reports *5*, 843–855.

Gat, U., DasGupta, R., Degenstein, L., and Fuchs, E. (1998). De Novo hair follicle morphogenesis and hair tumors in mice expressing a truncated beta-catenin in skin. Cell *95*, 605–614.

Gomez, C., Chua, W., Miremadi, A., Quist, S., Headon, D.J., and Watt, F.M. (2013). The interfollicular epidermis of adult mouse tail comprises two distinct cell lineages that are differentially regulated by Wnt, Edaradd, and Lrig1. Stem Cell Reports *1*, 19–27.

Grachtchouk, M., Pero, J., Yang, S.H., Ermilov, A.N., Michael, L.E., Wang, A., Wilbert, D., Patel, R.M., Ferris, J., Diener, J., et al. (2011). Basal cell carcinomas in mice arise from hair follicle stem cells and multiple epithelial progenitor populations. J. Clin. Invest. *121*, 1768–1781.

Hanahan, D., and Weinberg, R.A. (2011). Hallmarks of cancer: the next generation. Cell *144*, 646–674.

Harada, N., Tamai, Y., Ishikawa, T., Sauer, B., Takaku, K., Oshima, M., and Taketo, M.M. (1999). Intestinal polyposis in mice with a dominant stable mutation of the beta-catenin gene. EMBO J. *18*, 5931–5942.

Huelsken, J., Vogel, R., Erdmann, B., Cotsarelis, G., and Birchmeier, W. (2001). beta-Catenin controls hair follicle morphogenesis and stem cell differentiation in the skin. Cell *105*, 533–545.

Jaks, V., Barker, N., Kasper, M., van Es, J.H., Snippert, H.J., Clevers, H., and Toftgård, R. (2008). Lgr5 marks cycling, yet long-lived, hair follicle stem cells. Nat. Genet. *40*, 1291–1299.

Jensen, K.B., Collins, C.A., Nascimento, E., Tan, D.W., Frye, M., Itami, S., and Watt, F.M. (2009). Lrig1 expression defines a distinct multipotent stem cell population in mammalian epidermis. Cell Stem Cell *4*, 427–439.

Karin, M. (2009). NF-kappaB as a critical link between inflammation and cancer. Cold Spring Harb. Perspect. Biol. *1*, a000141.

Klaus, A., and Birchmeier, W. (2008). Wnt signalling and its impact on development and cancer. Nat. Rev. Cancer *8*, 387–398.

Kretzschmar, K., and Watt, F.M. (2012). Lineage tracing. Cell *148*, 33–45.

Kretzschmar, K., and Watt, F.M. (2014). Markers of epidermal stem cell subpopulations in adult mammalian skin. Cold Spring Harb. Perspect. Med. *4*, a013631.

Kretzschmar, K., Cottle, D.L., Schweiger, P.J., and Watt, F.M. (2015). The androgen receptor antagonizes Wnt/β-catenin signaling in epidermal stem cells. J. Invest. Dermatol. *135*, 2753–2763.

Kurokawa, I., Mizutani, H., Nishijima, S., Kato, N., Yasui, K., and Tsubura, A. (2005). Trichoadenoma: cytokeratin expression suggesting differentiation towards the follicular infundibulum and follicular bulge regions. Br. J. Dermatol. *153*, 1084–1086.

Leibl, H., Hutterer, J., Korschan, H., Schuler, G., Tani, M., Tschachler, E., Romani, N., Wolff, K., and Stingl, G. (1985). Expression of the Ly-5 alloantigenic system on epidermal cells. J. Invest. Dermatol. *84*, 91–95.

Liao, X.H., and Nguyen, H. (2014). Epidermal expression of Lgr6 is dependent on nerve endings and Schwann cells. Exp. Dermatol. *23*, 195–198.

Lim, X., and Nusse, R. (2013). Wnt signaling in skin development, homeostasis, and disease. Cold Spring Harb. Perspect. Biol. *5*, a008029.

Lim, X., Tan, S.H., Koh, W.L., Chau, R.M., Yan, K.S., Kuo, C.J., van Amerongen, R., Klein, A.M., and Nusse, R. (2013). Interfollicular epidermal stem cells self-renew via autocrine Wnt signaling. Science *342*, 1226–1230.

Lo Celso, C., Prowse, D.M., and Watt, F.M. (2004). Transient activation of beta-catenin signalling in adult mouse epidermis is sufficient to induce new hair follicles but continuous activation is required to maintain hair follicle tumours. Development *131*, 1787–1799.

Lowry, W.E., Blanpain, C., Nowak, J.A., Guasch, G., Lewis, L., and Fuchs, E. (2005). Defining the impact of beta-catenin/Tcf transactivation on epithelial stem cells. Genes Dev. *19*, 1596–1611.

Madisen, L., Zwingman, T.A., Sunkin, S.M., Oh, S.W., Zariwala, H.A., Gu, H., Ng, L.L., Palmiter, R.D., Hawrylycz, M.J., Jones, A.R., et al. (2010). A robust and high-throughput Cre reporting and characterization system for the whole mouse brain. Nat. Neurosci. *13*, 133–140.

Malanchi, I., Peinado, H., Kassen, D., Hussenet, T., Metzger, D., Chambon, P., Huber, M., Hohl, D., Cano, A., Birchmeier, W., and Huelsken, J. (2008). Cutaneous cancer stem cell maintenance is dependent on beta-catenin signalling. Nature *452*, 650–653.

Means, A.L., Xu, Y., Zhao, A., Ray, K.C., and Gu, G. (2008). A CK19(CreERT) knockin mouse line allows for conditional DNA recombination in epithelial cells in multiple endodermal organs. Genesis *46*, 318–323.

Merrill, B.J., Gat, U., DasGupta, R., and Fuchs, E. (2001). Tcf3 and Lef1 regulate lineage differentiation of multipotent stem cells in skin. Genes Dev. *15*, 1688–1705.

Morimoto, C., and Schlossman, S.F. (1998). The structure and function of CD26 in the T-cell immune response. Immunol. Rev. *161*, 55–70.

Müller-Röver, S., Handjiski, B., van der Veen, C., Eichmüller, S., Foitzik, K., McKay, I.A., Stenn, K.S., and Paus, R. (2001). A comprehensive guide for the accurate classification of murine hair follicles in distinct hair cycle stages. J. Invest. Dermatol. *117*, 3–15.

Niemann, C., Owens, D.M., Hülsken, J., Birchmeier, W., and Watt, F.M. (2002). Expression of DeltaNLef1 in mouse epidermis results in differentiation of hair follicles into squamous epidermal cysts and formation of skin tumours. Development *129*, 95–109.

Niemann, C., Owens, D.M., Schettina, P., and Watt, F.M. (2007). Dual role of inactivating Lef1 mutations in epidermis: tumor promotion and specification of tumor type. Cancer Res. *67*, 2916–2921.

Nijhof, J.G., Braun, K.M., Giangreco, A., van Pelt, C., Kawamoto, H., Boyd, R.L., Willemze, R., Mullenders, L.H., Watt, F.M., de Gruijl, F.R., and van Ewijk, W. (2006). The cell-surface marker MTS24 identifies a novel population of follicular keratinocytes with characteristics of progenitor cells. Development *133*, 3027–3037.

Page, M.E., Lombard, P., Ng, F., Göttgens, B., and Jensen, K.B. (2013). The epidermis comprises autonomous compartments maintained by distinct stem cell populations. Cell Stem Cell *13*, 471–482.

Pálmer, H.G., Anjos-Afonso, F., Carmeliet, G., Takeda, H., and Watt, F.M. (2008). The vitamin D receptor is a Wnt effector that controls hair follicle differentiation and specifies tumor type in adult epidermis. PLoS ONE *3*, e1483.

Peterson, S.C., Eberl, M., Vagnozzi, A.N., Belkadi, A., Veniaminova, N.A., Verhaegen, M.E., Bichakjian, C.K., Ward, N.L., Dlugosz, A.A., and Wong, S.Y. (2015). Basal cell carcinoma preferentially arises from stem cells within hair follicle and mechanosensory niches. Cell Stem Cell *16*, 400–412.

Powell, A.E., Wang, Y., Li, Y., Poulin, E.J., Means, A.L., Washington, M.K., Higginbotham, J.N., Juchheim, A., Prasad, N., Levy, S.E., et al. (2012). The pan-ErbB negative regulator Lrig1 is an intestinal stem cell marker that functions as a tumor suppressor. Cell *149*, 146–158.

Shimanovich, I., Krahl, D., and Rose, C. (2010). Trichoadenoma of Nikolowski is a distinct neoplasm within the spectrum of follicular tumors. J. Am. Acad. Dermatol. *62*, 277–283.

Silva-Vargas, V., Lo Celso, C., Giangreco, A., Ofstad, T., Prowse, D.M., Braun, K.M., and Watt, F.M. (2005). Beta-catenin and Hedgehog signal strength can specify number and location of hair follicles in adult epidermis without recruitment of bulge stem cells. Dev. Cell *9*, 121–131.

Snippert, H.J., Haegebarth, A., Kasper, M., Jaks, V., van Es, J.H., Barker, N., van de Wetering, M., van den Born, M., Begthel, H., Vries, R.G., et al. (2010). Lgr6 marks stem cells in the hair follicle that generate all cell lineages of the skin. Science *327*, 1385–1389.

Solanas, G., and Benitah, S.A. (2013). Regenerating the skin: a task for the heterogeneous stem cell pool and surrounding niche. Nat. Rev. Mol. Cell Biol. *14*, 737–748.

Takeda, H., Lyle, S., Lazar, A.J.F., Zouboulis, C.C., Smyth, I., and Watt, F.M. (2006). Human sebaceous tumors harbor inactivating mutations in LEF1. Nat. Med. *12*, 395–397.

Van Mater, D., Kolligs, F.T., Dlugosz, A.A., and Fearon, E.R. (2003). Transient activation of beta -catenin signaling in cutaneous keratinocytes is sufficient to trigger the active growth phase of the hair cycle in mice. Genes Dev. *17*, 1219–1224.

Watt, F.M., and Collins, C.A. (2008). Role of beta-catenin in epidermal stem cell expansion, lineage selection, and cancer. Cold Spring Harb. Symp. Quant. Biol. *73*, 503–512.

Watt, F.M., and Jensen, K.B. (2009). Epidermal stem cell diversity and quiescence. EMBO Mol. Med. *1*, 260–267.

Wielenga, V.J., Smits, R., Korinek, V., Smit, L., Kielman, M., Fodde, R., Clevers, H., and Pals, S.T. (1999). Expression of CD44 in Apc and Tcf mutant mice implies regulation by the WNT pathway. Am. J. Pathol. *154*, 515–523.

Wong, V.W., Stange, D.E., Page, M.E., Buczacki, S., Wabik, A., Itami, S., van de Wetering, M., Poulsom, R., Wright, N.A., Trotter, M.W., et al. (2012). Lrig1 controls intestinal stem-cell homeostasis by negative regulation of ErbB signalling. Nat. Cell Biol. *14*, 401–408.

Youssef, K.K., Van Keymeulen, A., Lapouge, G., Beck, B., Michaux, C., Achouri, Y., Sotiropoulou, P.A., and Blanpain, C. (2010). Identification of the cell lineage at the origin of basal cell carcinoma. Nat. Cell Biol. *12*, 299–305.

Youssef, K.K., Lapouge, G., Bouvrée, K., Rorive, S., Brohée, S., Appelstein, O., Larsimont, J.C., Sukumaran, V., Van de Sande, B., Pucci, D., et al. (2012). Adult interfollicular tumour-initiating cells are reprogrammed into an embryonic hair follicle progenitor-like fate during basal cell carcinoma initiation. Nat. Cell Biol. *14*, 1282–1294.

Zelger, B., Zelger, B.G., and Burgdorf, W.H. (2004). Dermatofibroma-a critical evaluation. Int. J. Surg. Pathol. *12*, 333–344.

SUPPLEMENTAL DATA ITEMS

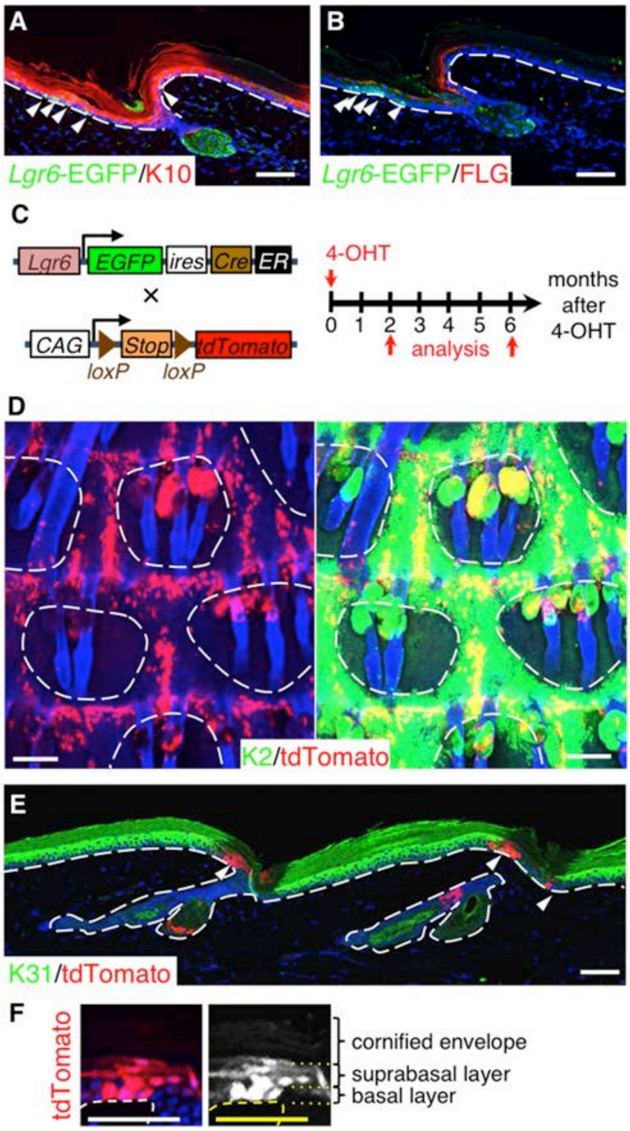

Figure S1 (related to Figure 1). *Lgr6*-expressing cells in the interfollicular epidermis give rise to the interscale differentiated lineage. (A, B) Tail skin sections collected from adult *Lgr6* KI mice stained with anti-EGFP (green), anti-K10 (red in A), anti-filaggrin (FLG; red in B) and counterstained with DAPI to label nuclei (blue). **(C)** Schematic representation of the genetic elements for lineage tracing during adult homeostasis and the experimental set-up. **(D)** Tail epidermal wholemounts collected from *Lgr6* KI/*R26R-tdTomato* mice collected 6 months after 4-OHT treatment, stained with anti-tdTomato (in red) and anti-K2 (in green). Wholemounts were counterstained with DAPI to label nuclei or with phalloidin to label F-actin (blue). Dashed lines demarcate IFE scales. **(E, F)** Tail skin sections collected from *Lgr6* KI/*R26R-tdTomato* mice twelve weeks after 4-OHT treatment, stained with anti-tdTomato (red) and anti-K31 (green) and counterstained with DAPI to label nuclei (blue). Dashed lines indicate epidermal-dermal boundary. Arrowheads denote LGR6^{+} cells or their progeny. F: higher magnification views of tdTomato+ IFE cells in (E). Scale bars: 100 µm (A, B, E, F), 50 µm (D).

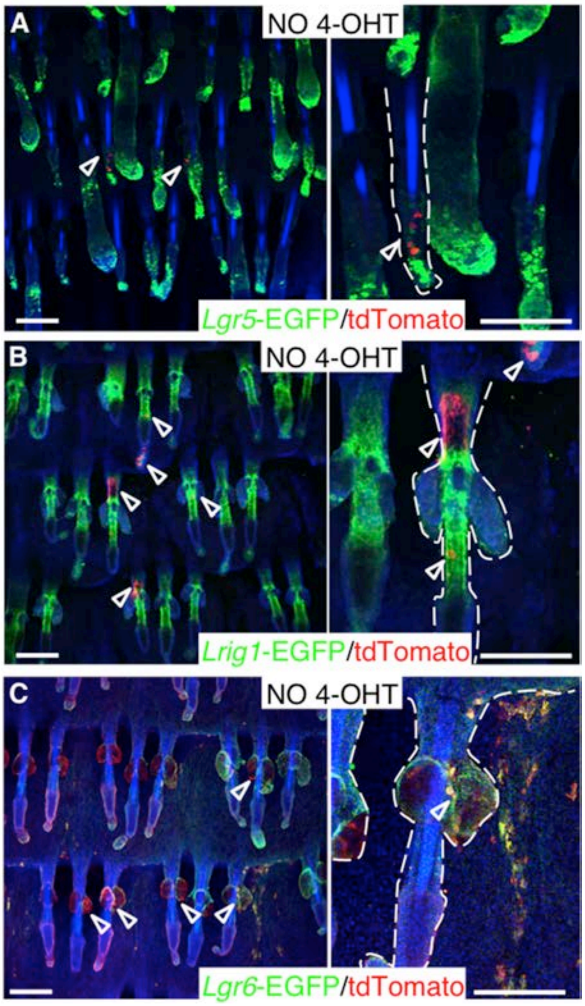

Figure S2 (related to Figure 1). Leakiness of KI reporters. (A) Detection of *Lgr5*-EGFP (green) and tdTomato (red) in tail epidermal wholemounts of acetone-only treated adult *Lgr5* KI/*R26R-tdTomato* mice. **(B)** Detection of *Lrig1*-EGFP (green) and tdTomato (red) in tail epidermal wholemounts of acetone-only treated adult *Lrig1* KI/*R26R-tdTomato* mice. **(C)** Detection of *Lgr6*-EGFP (green) and tdTomato (red) in tail epidermal wholemounts of acetone-only treated adult *Lgr6* KI/*R26R-tdTomato* mice. Dashed lines indicate pilosebaceous units. Arrowheads: tdTomato$^+$ cells. Scale bars: 100 µm.

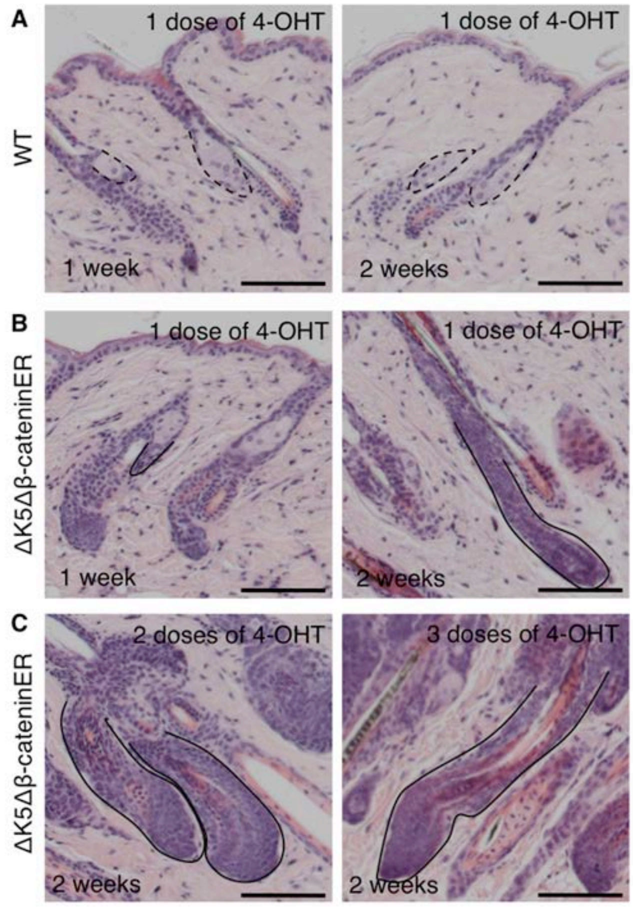

Figure S3 (related to Figure 2). Optimization of 4-OHT dose and tissue collection time points for ectopic hair follicle formation. (**A-C**) Wild-type (A) or ΔK5ΔNβ-cateninER transgenic mice (B, C) received one to three doses of 1.5 mg 4-OHT and tissue was collected one or two weeks later as indicated. Back skin sections were stained with H&E. Lines indicate ectopic HFs and dashed lines demarcate sebaceous glands. Scale bars: 200 μm.

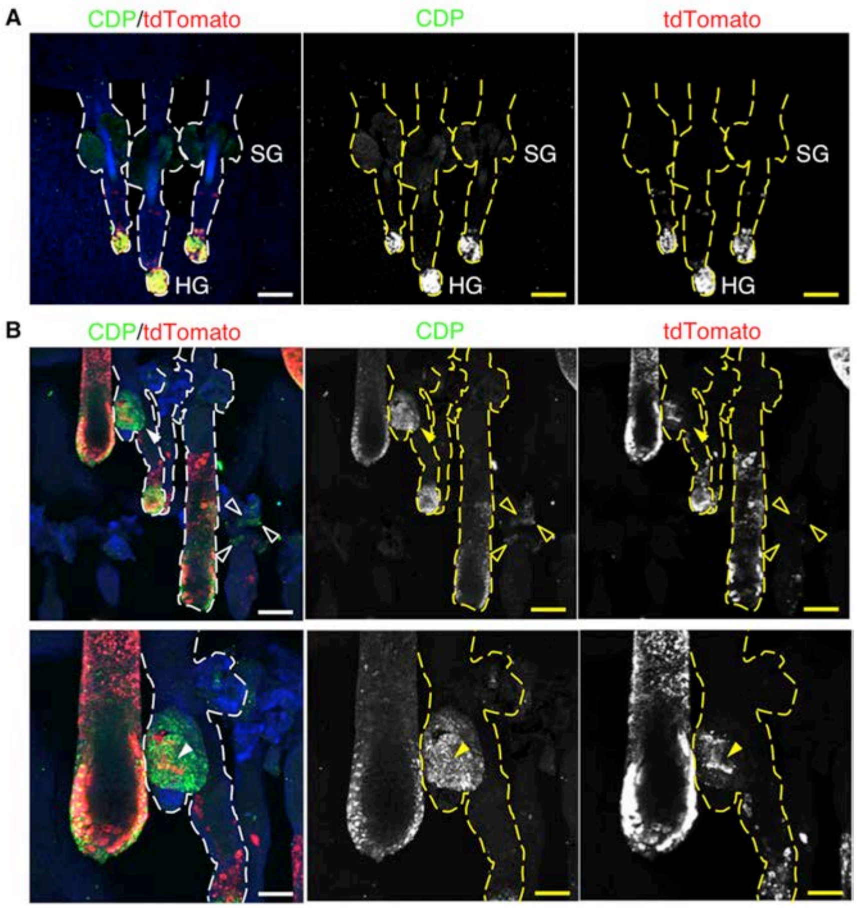

Figure S4 (related to Figure 2). Absence of CDP expression in control tail skin and example of tdTomato[+] progeny of *Lgr5*-expressing hair follicle stem cells. (A, B) Tail epidermal wholemounts collected one week after 4-OHT treatment and stained with anti-tdTomato (red) or anti-CDP (green), as indicated. Wholemounts were counterstained with DAPI to label nuclei or with phalloidin to label F-actin (blue). (A) *Lgr5* KI/*R26R-tdTomato* control mouse; (B) *Lgr5* KI/*R26R-tdTomato*/ΔK5ΔNβ-cateninER transgenic mouse. All panels in A show the same region of epidermis. All panels in B show the same region of epidermis, but lower panels are higher magnification views of upper panels. Single-colour images for individual markers are shown in grey scale. Dashed lines demarcate HF, SG and ectopic HFs. Individual ectopic HFs are indicated with closed arrowheads. Ectopic HF in IFE is shown with open arrowheads. Scale bars: 100 µm (A, upper panels in B), 50 µm (lower panels in B).

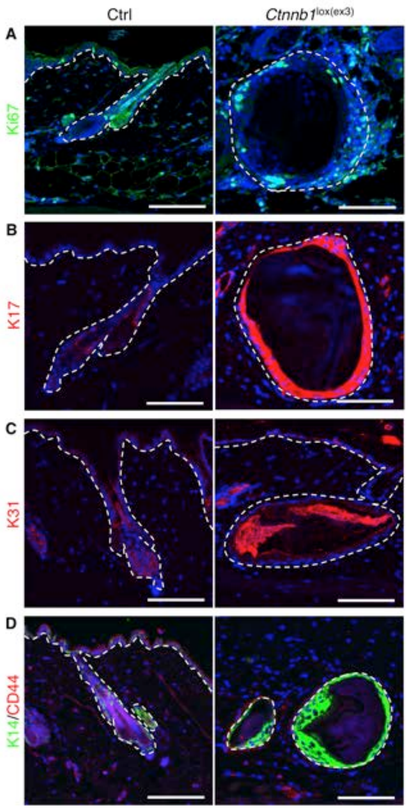

Figure S5 (related to Figure 3). Expression of Ki67, K17, K31 and CD44 following oncogenic β-catenin stabilisation in LGR5^{+} stem cells Paraffin sections of back skin collected from *Lgr5* KI/*R26R-tdTomato*/*Ctnnb1*$^{\text{lox(ex3)/+}}$ mutant and control *Lgr5* KI/*R26R-tdTomato*/*Ctnnb1*$^{+/+}$ (Ctrl) mice eight weeks after 4-OHT treatment, stained with antibodies against Ki67 (A), keratin 17 (K17), K31 (C), keratin 14 (D) and CD44 (D), counterstained with DAPI (blue). Dashed lines denote epidermal-dermal boundaries. Scale bars: 200 μm.

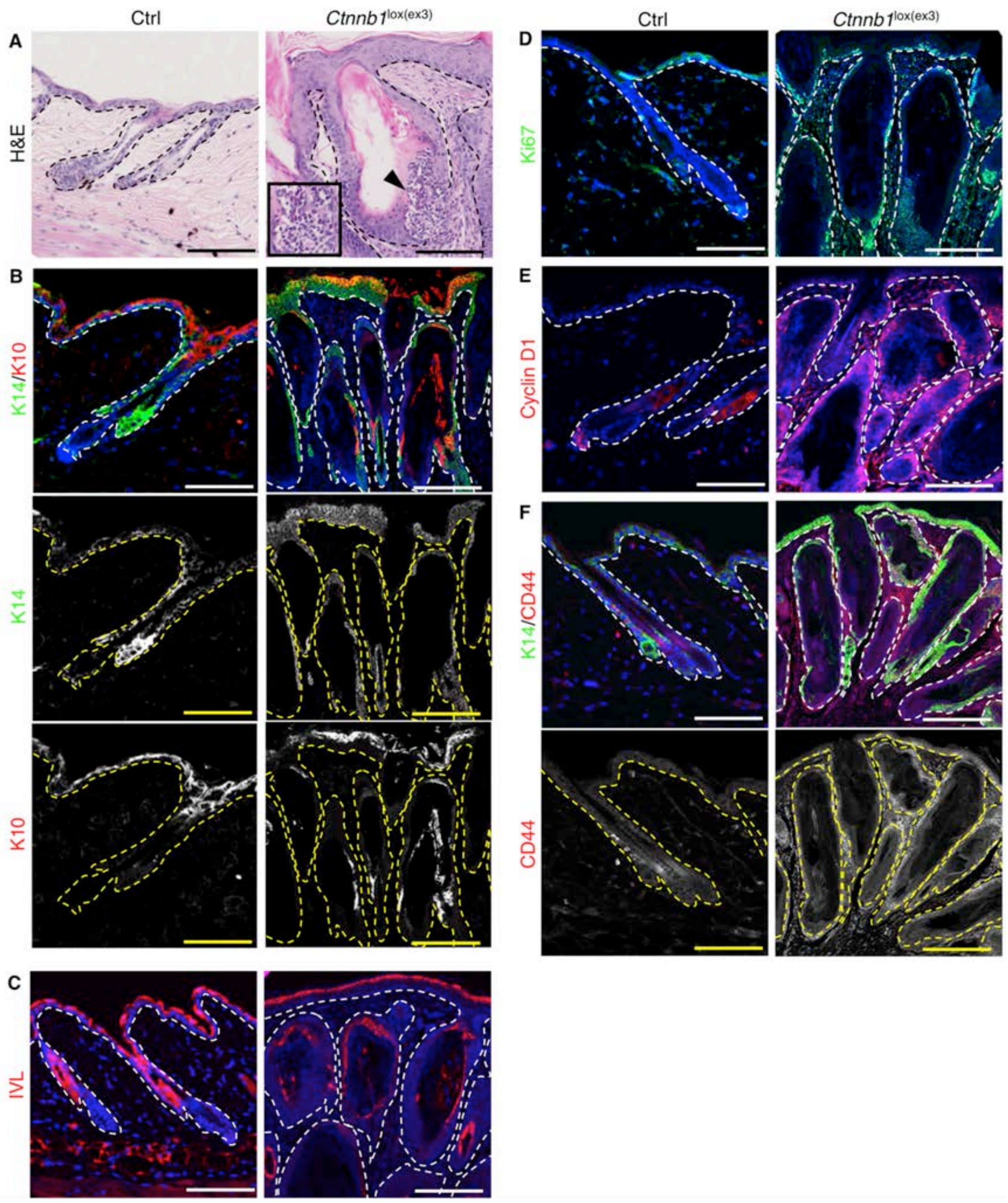

Figure S6 (related to Figure 4). Effects of oncogenic β-catenin stabilisation in LRIG1^{+} stem cells. Paraffin sections of back skin from *Lrig1* KI/*R26R-tdTomato*/*Ctnnb1*$^{lox(ex3)/+}$ mutant and control *Lrig1* KI/*R26R-tdTomato*/*Ctnnb1*$^{+/+}$ (Ctrl) mice four weeks after 4-OHT treatment, stained with H&E (A) or antibodies against K14 (green in B and F), K10 (red in B), involucrin (IVL; red in C), Ki67 (green in D), cyclin D1 (red in E), and CD44 (red in F), counterstained with DAPI (blue). Single-colour images for some markers are shown in grey scale, as indicated. Insert in (A) shows arrowed area at higher magnification. Dashed lines denote epidermal-dermal boundaries. Scale bars: 200 μm.

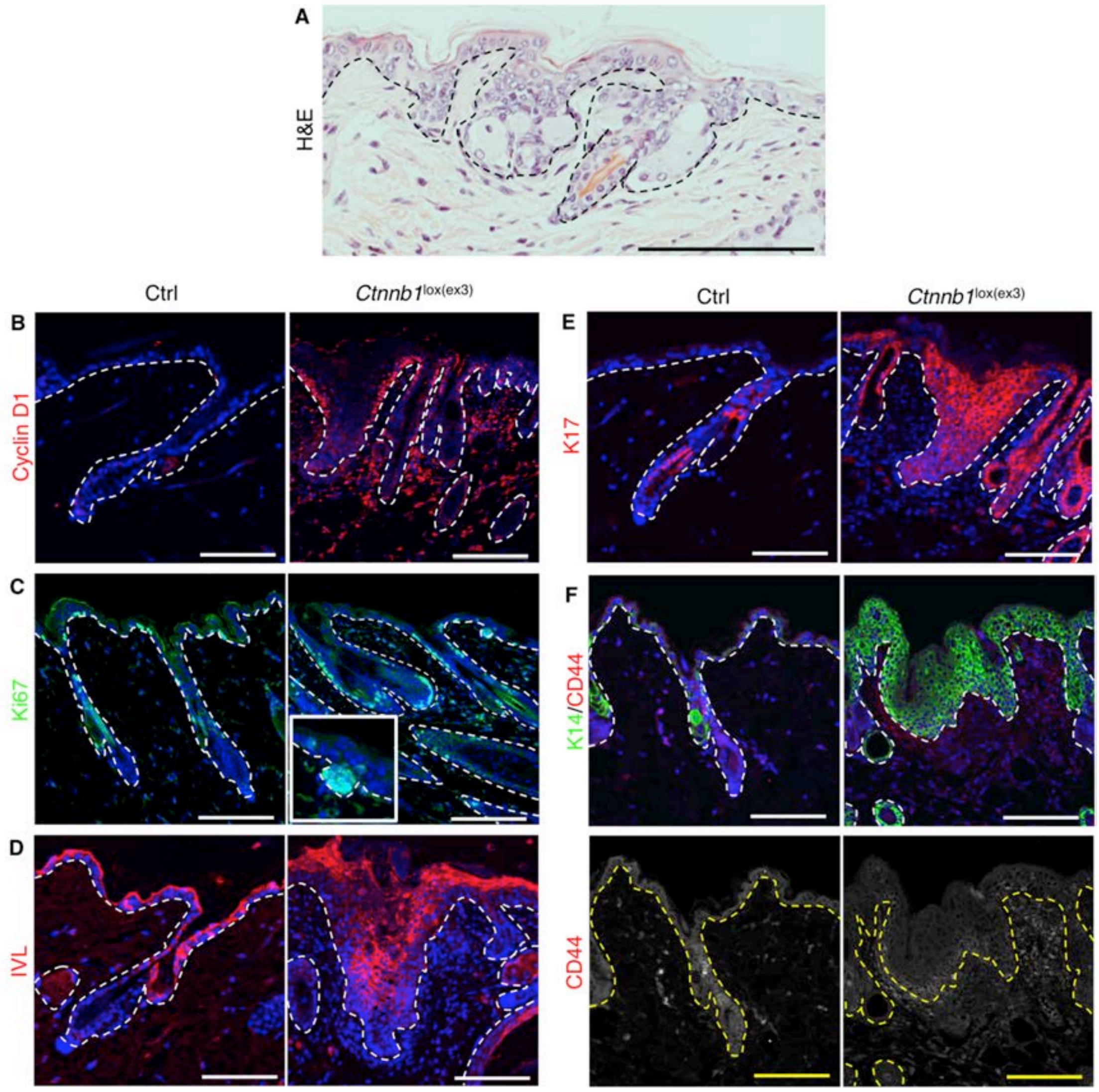

Figure S7 (related to Figure 5). Effects of oncogenic β-catenin stabilisation in LGR6^{+} stem cells. Paraffin sections of back skin collected from *Lgr6* KI/*R26R-tdTomato*/*Ctnnb1*$^{\text{lox(ex3)/+}}$ mutant and control *Lgr6* KI/*R26R-tdTomato*/*Ctnnb1*$^{+/+}$ (Ctrl) twelve (A) or eight (B-F) weeks after 4-OHT treatment, stained with H&E (A) or antibodies against cyclin D1 (red in B), Ki67 (green in C), involucrin (IVL; red in D), keratin 17 (K17; red in E), K14 (green in F), CD44 (red in F) and counterstained with DAPI (blue). Single-colour images for CD44 are shown in grey scale (F). Dashed lines denote epidermal-dermal boundaries. Insert in (B) shows IFE down growth (main panel) at higher magnification. Scale bars: 200 μm.

SUPPLEMENTAL EXPERIMENTAL PROCEDURES

Mice

For tdTomato lineage tracing and ectopic HF induction 7-9 week old adult mice were used. 1.5 mg 4-hydroxy-Tamoxifen (4-OHT; Sigma-Aldrich H7904) dissolved in 100 µL acetone (Sigma-Aldrich) was applied to the tail and shaved back skin. Activation of the *Ctnnb1* mutant allele was achieved by topical application of 1.5 mg 4-OHT dissolved in 100 µL acetone to shaved back skin of 7-9 week old adult mice. Both male and female mice were treated in the experiments and similar results were obtained.

Histology and immunohistochemistry

For immunohistochemistry, 5 µm sections were prepared and either stained with haematoxylin and eosin (H&E), stained following Herovici's protocol or subject to conventional antibody staining procedures as described elsewhere (Kretzschmar et al., 2015).

Primary antibodies used were: mouse anti-β-catenin (1:500; BD Bioscience 610153), goat anti-CD26 (1:50; R&D Systems AF1180), rat anti-CD44 (1:100; Abcam ab119863), rat anti-CD45 (1:100; eBioscience 14-0451), goat anti-CDP (1:100; Santa Cruz sc-6327), mouse anti-cyclin D1 (1:100; Santa Cruz sc-450), anti-F4/80 (eBioscience), rabbit anti-Filaggrin (1:100; Abcam ab24584), rabbit anti-GFP (1:500; Life Technologies A11122), chicken anti-GFP (1:500; Abcam ab13970), goat anti-GFP (1:200; Abcam ab6673), rabbit anti-involucrin (1:800; in-house ERLi-3), rabbit anti-K10 (1:100; Covance PRB-159P), mouse anti-K14 (1:1000; in-house LL002), rabbit anti-K14 (1:1000; Covance PRB-155P), chicken anti-K14 (1:1000; Covance SIG-3476), rabbit anti-K17 (1:300; in-house LT3TB4), guinea pig anti-K31 (1:100; Progen GP-hHa1), rabbit anti-Ki67 (1:100; Abcam ab16667), rat anti-Ki67 (1:300; DAKO M7249), rabbit anti-LEF1 (1:100; Cell Signaling #2230), goat anti-LRIG1 (1:100; R&D Systems AF3688), anti-NFκB (eBioscience), rabbit anti-RFP (1:1000; recognising tdTomato; Rockland 600-401-379), and rabbit anti-vimentin (1:100; Cell Signaling #5741). Antibody staining was visualised using appropriate species-specific secondary antibodies conjugated to Alexa Fluor 488, 555, 594 or 647 (1:300; Life Technologies). Slides were mounted using ProLong Gold anti-fade reagent (Life Technologies) containing 4',6-diamidino-2-phenylindole (DAPI; Sigma) as nuclear counterstain.

All fluorescent sections were analysed on a TCS SP5 confocal microscope (Leica) or an A1 confocal microscope (Nikon). All images of H&E and Herovici stained sections were taken on an Axiophot microscope with an AxioCam HRc camera (Zeiss) or on an A1 confocal microscope with a DS-Fi2 camera (Nikon). H&E stained tumour sections were scanned and analysed using Aperio ImageScope software (Leica).

SUPPLEMENTAL REFERENCE

Kretzschmar, K., Cottle, D.L., Schweiger, P.J., and Watt, F.M. (2015). The Androgen Receptor Antagonizes Wnt/beta-Catenin Signaling in Epidermal Stem Cells. The Journal of investigative dermatology.
